# Supplementary material for: Female adolescent sexual reproductive health service utilization concerns: A qualitative enquiry in the Tema metropolis of Ghana
Source: PLoS One. 2024 Feb 23;19(2):e0292103. doi: 10.1371/journal.pone.0292103 (PMC10890758; doi:10.1371/journal.pone.0292103)
Supplement: S1 File — (ZIP) [file pone.0292103.s001.zip › INTERVIEW 3 TRANSCRIPT.docx]

INTERVIEW 3 TRANSCRIPT

I; good afternoon, my name is Innes Agbenu. I am a student of the university of Ghana. I am doing an Mphil. As part of the programme, I am conducting research on how much or how well young adolescents utilize sexual reproductive health services. This afternoon, we are here to have an interview so we can share information on these reproductive health services. Thank you so much for agreeing to be part of the research. I will be asking a series of questions.

R; okay

Q; first of all, kindly tell me about yourself

R; I am a cool quiet girl, sometimes I am rude and sometimes quick tempered. I get quick tempered easily.

Q; how old are you?

R; I am18 years old

Q; your ethnic group?

R; I am an akan

Q; what religion do you belong to?

R; religion? Can you go further more?

Q; are you a Christian or a Muslim?

R; Christian

Q; Tell me about your educational experience?

R; go further

Q; I want you to tell me about the various schools you have attended

R; I went to primary school, junior high and now in senior high

Q; Tell me about your marital status?

R; I am not married

Q; Tell me about your work experience?

R; I have never worked before. I am a student

Q; do you have any source of income?

R; no

Q; Tell how do you get money when you need it?

R; from my parents

Q; Describe your living arrangements to me?

R; I live with my parents and my siblings

Q; so, considering your current age, will you categorise yourself as an adult or an adolescent?

R; I will say I am an adolescent

Q; as an adolescent, tell me some of the changes happening in your body?

R; when you reach the stage of adolescence, you become shy of the opposite sex, and you are quick tempered and you try to be arrogant

Q; so, with you personally, what are some of those things that are happening to you in terms of your body?

R; with me personally, I will say at first, in terms of my body, I was not developing breast but now I am developing breast. Also, at first, I was not having my menses but now I have started having me menses

Q; are there certain challenges that you face that you wish you could talk to someone about?

R; yes, that one is there

Q; are there some questions that run through your head that you wish to talk to somebody about?

R; yeah

Q; and the changes that are happening in your body, do you wish to talk to somebody about them?

R; I sometimes think not to talk about them but to keep them to myself. I also sometimes wish to talk to someone but I mostly like it when I keep it to myself

Q; is it easy to talk about those things?

R; not that easy

Q; so, on those days that you really need to talk, who do you talk to?

R; I mostly talk to my friends

Q; and who else?

R; apart from my friends, no one

Q; Okay. So, these things that happen to you, do feel like you need help?

R; yeah, sometimes, I need help

Q; and when you need help, what do you do?

R; if I need help, I tell my friends and if they have any advice for me, they tell me and when I think this is the advice that will for me, I take it or if not, I sit down to think about it and anything that comes into my mind, I do it if that will help me

Q; okay. At this age and being a female, are there certain needs that you have, that even when you are not sick, those needs are there and you wish you could get help from a health professional or from a hospital?

R; I will say no

Q; even if you are not sick, as a female adolescent with all the things happening in your body, you don’t sometimes need any professional help?

R; sometimes, it happens but sometimes, it doesn’t

Q; so, when that need arises, what do you do?

R; hmmm, when it happens, hmmm, it is either I find a way; maybe someone has experienced that before, I ask the person so the person can help me. Maybe, what I am facing, the person once had it and it is related to mine, so I ask the person what she did, then the person can tell me

Q; do you know anything about sexual reproductive health?

R; yeah

Q; tell me what you know about sexual reproductive health

R; sexual reproductive to me, it means having sex and protecting yourself from other diseases

Q; and then what else?

R; also, you can abstain yourself from sexual intercourse

Q; where did you hear about sexual reproductive health?

R; in social studies and teaching. Sometimes too, on television when they talk about adolescent, I try to learn from that

Q; tell me some of the things you know can happen to you if you engage in sexual activity

R; you can get pregnant and you can attract diseases into your body

Q; Tell me what you know of adolescent health clinics located within Tema?

R; I don’t know anything about that

Q; not even in your area?

R; no

Q; you mean you don’t know of anywhere that as an adolescent, with all the things you have told me that you can go and seek help?

R; no

Q; okay. In your own expectations or thoughts, what are some of the services that you think an adolescent sexual reproductive health center will provide?

R; I think they should they should at least, sometimes help, some adolescents feel shy when talking to people in that way. So, they should find a way of advising them, trying to engage them in talks, if not, they will not come to you

Q; Tell me what you know about contraceptives?

R; for the contraceptives, what I know is you taking it for sex or after sex. Some also, when you get pregnant, you can take some to abort the child

Q; where did you get the information about contraceptives?

R; on television, I sometimes read about it, and also, from friends

Q; alright, thank you. You told me that as a female at this age, if you engage in sexual activity, you can get pregnant or get some diseases. Now, as an adolescent, do you know of anywhere that you can go and get help to prevent any of these occurrences?

Q; okay, before you try to have sex, you need to seek advice from an elderly person before you go for it. But some people, maybe it is a mistake and it happen, you can go to an elderly person and tell them, maybe she can help you in some way

R; so in what ways do you think you can prevent pregnancy and sexually transmitted diseases?

R; for me, abstinence from sex and using of condoms

Q; well, so, in case any of these things, the pregnancy or diseases related to sex occurs, where do you know that you can go and get help?

R; hmmm, for the pregnancy, I don’t know but like for me, I will go to my parents and whatever they will do for me, they will. For the disease too, the same thing. I will tell my parents. They will try and get help somewhere for me to go

Q; do you know of any hospital, clinic or health center that you can go to yourself and talk about these things?

R; no

Q; do you experience any issue with your menses

R; oh no. the day my menses doesn’t come is when I change my environment but after two or three months, it starts coming

Q; what of other issues like pain or the amount of bleeding?

R; the pain is a lot but the bleeding is not too much. It lasts for four to five days

Q; what do you do when the pain comes?

R; I take medicine to cool down the pain and I relax a bit

Q; where do you get the medicine from?

R; sometimes, my mum gives to me and it is not always that I take the medicine because when you take the medicine too much, the body can adjust to it so sometimes, I just sleep so that it will cool sown

Q; have you ever gone to the hospital to get help for the menstrual pains?

R; no ooo

Q; why?

R; hmm, the time is a little

Q; is it only about time?

R; okay, it has not occurred to me or come to my mind to do that

Q; but are you aware you can go to the hospital to talk to somebody?

R; that one, I know

Q; so why don’t you go?

R; hesitates for a while……, I don’t have a reason but I think it is good. Maybe going to get advice will help you know what to do

Q; so as a female adolescent too, I guess you sometimes use public washrooms and other things. Have you ever gotten any infection of the genital tract?

R; I sometimes get “white” but before it becomes severe, I use joy ointment and panty liner so that it will go fast. I don’t want it to spread

Q; apart from joy ointment, what else have you used for the infections before?

R; since I use the joy ointment when it is coming, it doesn’t spread. So, I think there is no need for me to go to the hospital

Q; so you don’t go to the hospital because you don’t see the need to go?

R; yes

Q; how useful is the NHIS to you as an individual?

R; to me, it helps by taking care of some part of the cost of medicine

Q; do you know whether it covers the cost of these sexual reproductive health services we are talking about?

R; I don’t know

Q; when you visit the hospital, who pays?

R; my parents

Q; have you ever been to the hospital?

R; yes

Q; for what?

R; I have been to the hospital for a surgery to be done before

Q; what kind of surgery?

R; I got hurt on my leg. A branch of tree fell on my leg so we went to the hospital and they said they need to put some metals into my bones to adjust them. That was the last time I went to the hospital

Q; does the availability of money or money not being available influence your decision to go to the hospital?

R; for me, I don’t like the hospital so me I don’t think about the money but like my family, if you need to go to the hospital, my parents will pay for it

Q; why don’t you like the hospital?

R; hmmm, the injection. I don’t like injections

Q; what else don’t you like about the hospital?

R; the medicines

Q; is it just about the medicines and injection? What about the people who work there?

R; for me I have never experienced a bad treatment from a nurse

Q; so all the time you met nice nurses at the hospital?

R; yes

Q; have you ever used a contraceptive?

R; no

Q; why?

R; no reason. Maybe because I have not yet engaged in sex

Q; what do you think about sexual reproductive health services for adolescents?

R; for me, what I will say is that as an adolescent, not all the things that your mum has told you that she may know all but we getting to the health center can help us know more

Q; so do you think these services are important?

R; yes, they are important

Q; do you think that there is enough information about these services so that you can get to use them when you want to?

R; no, I think there is not enough information. For some of us, not all of us know about it. They need to get more information out and communicate with adolescents so that they can know more about it. Because if they do not know about it, they will end up shunning it

Q; from the conversations you have with your friends, do you think they also know about these services?

R; for that one, I think they don’t know

Q; what do you think should be done about these services so that we encourage adolescents to use them more?

R; maybe you go to schools and organize the girls and try to talk to them about these things. Maybe you can also bring someone who has experienced that before and the person will come and explain to them. I think that will help them to understand better

Q; is there any other thing you will want to share with me concerning adolescents and sexual reproductive health services?

R; hesitates……

Q; you have been talking to your friends, I guess there are a lot you can share with me. Maybe some recommendations

R; for me, what I know is that some go for sexual activity and they don’t take the contraceptives but they are okay. No disease and no pregnancy. Some of them too, they are in the relationship but never engaged in sex and some also do out for the money

Q; that will be all for now. Thank you very much
