# Supplementary material for: Female adolescent sexual reproductive health service utilization concerns: A qualitative enquiry in the Tema metropolis of Ghana
Source: PLoS One. 2024 Feb 23;19(2):e0292103. doi: 10.1371/journal.pone.0292103 (PMC10890758; doi:10.1371/journal.pone.0292103)
Supplement: S1 File — (ZIP) [file pone.0292103.s001.zip › Interview eight transcript.docx]

Interview eight transcript

Interviewer; good afternoon. My name is Innes Agbenu. I am a second-year masters’ student from the university of Ghana school of nursing and midwifery. I am conducting a research on the utilization of sexual reproductive services among female adolescents within the Tema metropolis. As we discussed earlier, we are going to have an interview this afternoon where I will be asking you questions and you will be providing responses based on the topic we will be discussing. Thank you once again for agreeing to take part in the study.

Respondent; okay

I; to start with, can you tell me about yourself?

R; my friends call me mad lion. I study and live in Tema. I live at guest in. I live with my mother who is a single parent. I am dark in complexion. I come from kumawu in the Ashanti region. I am very cool with people and I like to mingle

I; are you married?

R; no

I; are you employed?

R; please I don’t understand

I; do you work?

R; yes. I used to sell things by myself like chains, watches

I; where do you sell them at?

R; sometimes in my area and sometimes in school

I; do you still sell them?

R; yes

I; considering your current age, will you consider yourself as a child, an adolescent or an adult?

R; I will consider myself as an adolescent

I; what are some of the experiences associated with the adolescent period for you as an individual?

R; I have some physical and emotional changes. Some of them are anger, shyness, growing of pubic hair, breast and we the girls too, we menstruate

I; do you menstruate currently?

R; yes

I; with all these changes taking place, do you at times feel like you need someone to talk to?

R; yes, I talk to my mum and she is the one who buys my pad for me

I; is it easy for you to discuss such things with your mother?

R; not really

I; why?

R; some of those things, for example, I feel pains during my menses and my menses usually comes a week after my mother’s so she already knows my time to menstruate and so, there is nothing to discuss with her

I; at this age, are there times that even when you are not seek you feel you have certain needs that you wish you could get help for from the hospital or a healthcare provider?

R; yes. Sometimes, we have to got to the hospital to have check ups. This is because at times for instance, you can use a sharp knife or blade which you may not know is infected. You therefore have to go to the hospital to check how your blood system is working because you may not know whether you are HIV positive or negative. And also, check for other viruses that have come to exist so that we can get treatment

I; so, do you go to the hospital to check yourself?

R; yes, sometimes, I used to go. I used to donate blood to people so they check before I donate to make sure that everything is fine

I; you say you donate blood?

R; yes

I; how long have you been donating blood?

R; for the past 3 years. I started since I came entered form one. Every year, we organise blood donation at korle bu and general hospital where we donate blood for those who are in need of blood but don’t have

I; okay, that’s a good one. Tell me what you know about sexual reproductive health

R; sexual reproductive health is using substances to protect yourself from any form of diseases that come from sex such as using condoms and family planning

I; where did you hear about sexual reproductive health?

R; I listen to the news a lot. I also watch programmes on Tv that talk about how women protect themselves from infections

I; what are some of the things you know can happen to you when you engage in sexual activities?

R; there can be teenage pregnancy because of my age and I can drop out of school. As for me if I should get into that situation, my mum will not take care of me again. She will say it means I am of age and can take care of my self that is why I am going in for a man and having sex at the same time. I can also get HIV from that because I don’t know the person I am going to sleep with

I; are you aware of adolescent sexual reproductive health centres located within Tema?

R; I know of the nurse in my school. She gives us education on how to protect ourselves and abstinence from sex. Sometimes too, when you visit clinics in the community, they try to talk to you about what an adolescent must do like things about sex, the causes and the problems they can give

I; have you visited any of those clinics before?

R; not actually

I; why?

R; because for me, I leave home early and I am mostly not home except for Saturdays and Sundays but I know people who go

I; you have friends who go there?

R; yes

I; have they shared with you what their experiences have been?

R; you know that some of these adolescents, no matter what you tell them, they will not listen so they tell them that if you are in that relationship already (having sex), then you can do family planning or you get condoms to protect yourself against diseases

I; does that mean you know of some of the services they provide there?

R; yes

I; what are some of them?

R; they give advise and family planning

I; okay, what do you know about contraceptives?

R; they are medicines that are given to a person so that they will not get pregnant

I; what are some of the contraceptives you know?

R; V firm

I; and which other one?

R; I cannot remember the rest

I; are you sure?

R; yes

I; okay, as we go along do let me know when you remember any other one. Now, in case you ever engage in sex, do you know ways in which you can prevent pregnancy or infections and diseases?

R; to prevent infections, you must protect themselves by using condoms. As for infections, people do take medicine to prevent it. You can take medicine before sleeping with your husband or your guy(boyfriend). For the HIV, you can use condom to prevent it

I; so, in case the pregnancy happens, or you get any of the infections, do you know what you can do about it?

R; if you get the infections, you have to seek treatment so that you will not die early. Like the HIV, you will need to get treatment so that you don’t die early. People think that once you get HIV, you will die early but they have some medicine that you can take so that you can live longer

I; have you ever experienced any infections with your reproductive system which is not necessarily from sex?

R; yes

I; can you share with me your experience?

R; I had white (candidiasis). The white, some is natural and some too, some people get it more and it is due to infection. I remember my friend had the white and she used to go to the clinic for treatment everyday. It used to come in a yellowish color. As for the natural, it sometimes comes during the menses and goes but as for her own, anytime at all, it comes

I; have you experienced any of the white yourself before?

R; the natural one, yes

I; what did you do about it?

R; they always tell me it is natural so you have to use water to wash it.

I; who said it is natural?

R; that is what they told me

I; who told you that?

R; people say that

I; who are those people?

R; my friends. They say it is normal. Like when you tell them that; “the place is itching me ooo”, they reply that “as for this one, it is normal”

I; so it does itch too?

R; yes, the itching is there and sometimes the smell too

I; what do you do for the itching to go?

R; I for instance, it comes more before and during the menses and then it goes after that. I just use water. They say water so I just use water

I; okay.

I; do you use the national health insurance?

R; no

I; why?

R; I have it but since 2013, it got expired and since then, I haven’t renewed it

I; is it important to you?

R; yes, it is important because it is not everyday that there will be money to visit the hospital for treatment but when you use the health insurance, it reduces the money taken at the hospital. Maybe taking money to pay for some of the drugs is difficult so it will take care of part of the healthcare

I; if you have to go to the hospital, does the fact that you have money or does not have money influence your decision to go?

R; once you have the health insurance, you still have to go to the hospital. You don’t know the actual money or you don’t know whether the health insurance can cover all your sickness cost so you have to go

I; do you know whether the health insurance covers these sexual reproductive health services we are talking about?

R; yes, I know it does. When my friend visited the clinic for the treatment of the white, I asked her whether they take money from her and she said she only did the card for 2 cedis but the health insurance covered the cost of treatment

I; do you think it makes things easy?

R; yes, it makes you to save a lot

I; have you ever been to the hospital for any treatment related to sexual reproductive health?

R; no, I am very strong

I; in case any of that happens, what will you do?

R; I will seek for treatment

I; at where?

R; at any health center

I; and if should get pregnant too?

R; as for pregnancy, I will give birth because according to the Bible, it is not good to shed blood

I; have you used any contraceptive before?

R; no

I; why?

R; I wouldn’t bother myself to engage in sex so I don’t need a contraceptive

I; how come you haven’t visited the adolescent sexual reproductive health center as your friends have been doing?

R; I can say that I used to go but now I talk to my school nurse anytime I have difficulties and she always gives me advise and tell me what to do for that problem to stop

I; how was the experience when you went?

R; it was a good one

I; did you learn a lot?

R; yes

I; will you go there again?

R; I will like to go there again but not at the clinic

I; why?

R; I don’t like the behaviour of some of the nurses over there. Anytime you visit that place, they always think something about you. I once went there for the treatment of malaria and one of the nurses asked me that “are you pregnant”? and my mother asked which pregnancy she is talking about. They have to learn how to talk to us when we go there. I don’t like how they talk. Because of that, people are afraid to go there. Sometimes when you go, they don’t have time for you. They will be doing things on their mobile phones. They have to stop that. I will prefer my personal doctor or nurse that will have time for me

I; in general, what do you think about sexual reproductive health services for adolescents?

R; there should be improvements because some of the adolescents, we are afraid to go there. The health insurance should cover more of the services we go there for

It is good for us to go there because it helps us to learn things about ourselves and some of the nurses also motivate us

I; so what are some of the improvements you think should be done?

R; they should build more of those clinics especially in the rural areas especially in the villages

I; do you have any other information to share with me concerning adolescent sexual reproductive health?

I; I will say that they should provide more education for the adolescents and also about the contraceptives, how they can protect themselves against teenage pregnancy

I; okay, thank you so much for talking to me
