# Supplementary material for: Female adolescent sexual reproductive health service utilization concerns: A qualitative enquiry in the Tema metropolis of Ghana
Source: PLoS One. 2024 Feb 23;19(2):e0292103. doi: 10.1371/journal.pone.0292103 (PMC10890758; doi:10.1371/journal.pone.0292103)
Supplement: S1 File — (ZIP) [file pone.0292103.s001.zip › Interview eleven transcript.docx]

Interview eleven transcript

Interviewer; good afternoon

Respondent; good afternoon

I; my name is Innes Agbenu. I am a second-year master’s student from the university of Ghana. I am conducting a research on how young women within the Tema metropolis use sexual reproductive services. We will be having a discussion on that this afternoon and I will be recording this discussion which we will later sit down to make meaning out of. Thank you once again for accepting to be part of the research

R; okay, you are welcome

I; first of all, kindly tell me about yourself

R; my name is Bernice and I am seventeen years old. I don’t know what to say again

I; okay, I will ask you questions about yourself then. What ethnic group do you belong to?

R; I am an Ewe

I; what is your religion?

R; Christian

I; where have you gone to school up to?

R; after JHS, I continued to SHS. That is where I am now

I; are you married?

R; no

I; do you work?

R; no

I; do you have any source of income?

R; no

I; when you need money, how do you get it?

R; I call my brother or my father to send me money

I; whom do you live with?

R; my sister

I; is she older or younger than you?

R; she is older

I; do you live with her alone?

R; no, I live with her and her children

I; with your current age, will you consider yourself as a child, an adolescent or an adult?

R; an adolescent

I; why do you consider yourself as an adolescent?

R; because the age of seventeen, you need to know much about sexual intercourse and how to prevent it

I; as an adolescent, what are some of the changes happening in your body?

R; changes in my breast, they have grown bigger and I have also grown pubic hair

I; are those the only changes you have noticed?

R; yes

I; you mentioned that at this age, there are things you need to know about sexual intercourse and all that. Are you able to talk to your sister about these things?

R; no

I; why?

R; because at first, I used to hear those things but I never involved myself in them

I; do you have questions about sexuality that you wish you could talk to someone about?

R; yes, I ask my friends and some of them tell me that if you have sex at this time, you can get pregnant and since we are still students, it will not be good to do that

I; as an adolescent and a female, there may be times that even though you are not sick, you may have certain issues that you wish to talk to a healthcare provider or visit the hospital. Do you sometimes experience such things?

R; yes, sometimes I experience lower abdominal pains and I tell my friends about it. They tell me that I can take in paracetamol for it so I take in paracetamol

I; where do you get the paracetamol from?

R; I buy it

I; from where?

R; from the drug store

I; have you considered going to the hospital?

R; when I take the paracetamol, it reduces the pain a little

I; what else do you experience in relation to your reproductive health?

R; sometimes, I get a certain infection like white

I; and when that happens, what do you do?

R; with that one, the toilet we used to go, when I use it, the heat from the toilet makes me to get white. When it comes, I use joy ointment

I; how do you use it?

R; they wrote it on it that you put it in cotton and insert it into the vagina so I do that

I; how did you hear about this joy ointment?

R; I heard it from a friend. She told me it is good so I also use it

I; tell me about sexual reproductive health

R; what I know about it is that when you are going for sex, you need to protect yourself with condom and those things

I; where did you get this information from?

R; I have been hearing it from my friends

I; what else have you heard about sexual reproductive health?

R; that’s all I know

I; at this age, what are some of the things that can happen to you when you engage in sex?

R; maybe you get infection like HIV/AIDS or gonorrhea. Sometimes, as a teenager, you may get pregnant and, on the way, it will make things difficult for you

I; what things will be difficult?

R; if you get pregnant, maybe the man will not look after the baby and you will start suffering

I; do you know any centers within hospitals around that provide sexual reproductive health services for adolescent?

R; no, I don’t know anything about that

I; have you heard about places that adolescents can go for services related to their sexual reproductive health?

R; I have heard about it but I have never been there before

I; where did you hear about it?

R; sometimes, I sit among grownups and I heard them discussing it

I; what did they say about where these places are located?

R; I don’t really know

I; did you hear them talk about the services that are provided there?

R; I didn’t hear that one too

I; do you know of ways in which one can prevent pregnancy and those sexually transmitted infections you told me about earlier?

R; I know of only one prevention

I; and what is that?

R; by using condom

I; apart from condoms, what else?

R; I don’t know of any other

I; have you heard about contraceptives?

R; yes

I; share with me what you know about contraceptives

R; please I have not heard about it before

I; have you heard about medicines that people take to prevent pregnancy?

R; like family planning?

I; yes. What have you heard about those?

R; I have heard that those medicines when you take them and you have sex, you will not get pregnant. There are some tablets too that after the sex, when you take them, you will not get pregnant.

I; share with me the types of family planning you know about

R; like Lydia, after having the sex, you can take it and it will protect you

I; have you taken any of them before?

R; no

I; why?

R; nothing. I am not having sex

I; in the event that you engage in sex and get pregnant or get any of the infections, what will you do about it?

R; with the pregnancy, some people will abort and some will also give but. With the infections too, you will go to the hospital to get treatment

I; as an adolescent, if you get pregnant, do you know places you can visit in the hospital to get help?

R; as for me, I have heard about them but I have never been there

I; do you know that places also exist to provide education and services to adolescents on their sexual reproductive health?

R; maybe

I; do you have health insurance?

R; yes

I; do you use it?

R; no

I; why?

R; I have not been going to the hospital

I; don’t you fall sick? You mentioned you have white, why haven’t you gone to report that at the hospital?

R; when I use the joy ointment, it reduces it a little

I; meaning it doesn’t go entirely?

R; yes

I; so why haven’t you reported to the hospital with it?

R; like, I don’t like hospitals

I; why?

R; nothing

I; have you had any bad experience at the hospital before?

R; I have not been there before

I; if you haven’t been there before, why don’t you want to go there?

R; nothing

I; okay, I see

I; is your decision not no go to the hospital influenced by money?

R; no, it is not about money

I; is it that you are shy?

R; no

I; you just don’t want to go to the hospital?

R; yes

I; in as much as you don’t want to go the hospital, don’t you at times have any health needs that you wish to take to the hospital?

R; sometimes, my body used to itch. But I was told it is due to the food I eat and the pomade I am using so I think I need to change that thing

I; does that mean visiting the hospital never came to mind?

R; no

I; have you ever used any contraceptive?

R; no

I; has there ever been any occasion that you visited the hospital for any service?

R; no. if a problem comes, I will go

I; from what we discussed, you mentioned some things like menstrual pains and white, and from the discussion we have had so far, these things are a problem for you. You even did some things like buying some medications to help solve the problem. Is there any reason why you haven’t thought of going to the hospital?

R; it is not anything

I; is it that you are not aware you can visit the hospital with these problems?

R; I know I can go.

I; okay, if you know, then why don’t you go?

R; I don’t think it is serious enough for me to go to the hospital

I; what do you think about sexual reproductive health services for adolescents?

R; to protect yourself, not to engage yourself in activities that will harm you

I; do you think they are important?

R; yes

I; do you think there is enough information on those things that gives you confidence to use them?

R; I don’t think so

I; what do you think should be done to encourage you to use these services?

R; to educate us more so that we will know more about it so that we know what to do at the right time

I; do you have any other information for me pertaining to what we have been discussing so far?

R; no

I; okay. Thank you so much for your time

R; you are welcome
