# Supplementary material for: Female adolescent sexual reproductive health service utilization concerns: A qualitative enquiry in the Tema metropolis of Ghana
Source: PLoS One. 2024 Feb 23;19(2):e0292103. doi: 10.1371/journal.pone.0292103 (PMC10890758; doi:10.1371/journal.pone.0292103)
Supplement: S1 File — (ZIP) [file pone.0292103.s001.zip › INTERVIEW FIVE TRANSCRIPT.docx]

INTERVIEW FIVE TRANSCRIPT

Interviewer; good morning, how are you?

Respondent; good morning, I am fine

I; my name is Innes Agbenu, I am a second-year master’s student of the university of Ghana school of Nursing and Midwifery. As part of the programme, I am conducting a research on the utilization of sexual reproductive health services among female adolescents in the Tema metropolis. As we discussed earlier, this morning, we are here to have an interview where we will be discussing issues concerning sexual reproductive health services of female adolescents. I want to thank you once again for accepting to be part of the study and for agreeing to grant me this interview. Thank you

R; you are welcome

I; okay, first of all, can you please tell me about yourself?

R; I am a student and I live at Tema, community twenty-five. I am a girl.

I; how old are you?

R; sixteen

I; Which ethnic group do you belong to?

R; I am Ga-adangbe

I; what is your religion?

R; Christianity

I; what is your level of education?

R; secondary

I; are you married?

R; no please

I; tell me what you do for work?

R; I am a student

I; what is your source of income?

R; I don’t have any source of income

I; how do you get money when you need it?

R; from my parents

I; whom do you live with?

R; I live with my parents

I; considering you current age that you mentioned to me, will you categorise yourself as a child, an adolescent or an adult?

R; I am a teenager

I; is that to mean that you are an adolescent?

R; yes

I; why will you classify yourself as an adolescent?

R; I know most of the things that are not good for me and those that are good, I am in my cycle too so I am supposed to be an adolescent

I; can you share with me the things that are happening in your body as an adolescent?

R; I see my menstrual cycle; I have different looks when I am in my menses and I have different looks too when I am out of it. Sometimes too, I get tired for no specific reason. I also get easily provoked

I; with these things that are happening in your body, do you at times feel like talking to somebody about them?

R; yes, I talk to my mum

I; apart from your mum, do you talk to any other person?

R; she always tells me she is my best friend

I, so, what do you discuss with your mum?

R; everything happening in my body

I; and does she provide all the answers that you need?

R; yes

I; with your current age and the things happening in your body, do you think you have any health needs?

R; no please

I; what I mean is that, you may not necessarily be sick, but you may have some issues that you wish you can get help for from a healthcare provider?

R; I don’t think so

I; can you share with me what you know about sexual reproductive health?

R; actually, I don’t have a fair idea about it but what I know is that it is about your sexual life and how you need to take care of yourself

I; can you tell me the source of this information?

R; I hear my friends talking about it

I; apart from your friends, where else?

R; my parents too, sometimes

I; anymore sources?

R; no. I am actually an introvert so I don’t really mingle with others

I; at this age and with everything happening in your body, can you tell me some of the things that can happen to you when you engage in sexual activity?

R; I can get sicknesses like HIV and other things. I can get gonorrhea too

I; what else can happen?

R; I can get destroyed because if I should have sex without protecting myself and I get this virus, my life is going to be miserable. Even though I may be given some drugs to maintain my lifespan but I will not be okay

I; are you aware of any adolescent health clinics situated in Tema?

R; I am not sure

I; have you heard of any place in Tema that provides help for adolescents in relation to their sexual reproductive health issues?

R; I think Port medical center does that and New Crystal hospital at community eight too

I; what are some of the things you know they do for adolescents?

R; they come to we the adolescents and talk to us about how to take care of our body, to abstain from certain things that will make our lives short

I; can you tell me what you know about contraceptives?

R; the only thing I know about it is after sex, it is taken and it prevents pregnancy

I; what do you know about types of contraceptives?

R; I don’t know about them

I; do you know of places you can go to get help to prevent pregnancy?

R; yes

I; tell me some of these places

R; the hospital, you can talk to your doctor. When you don’t want to be pregnant again, I hear they will sew your something and that will block the sperm from entering your womb and prevent pregnancy

I; you mentioned earlier that there are some diseases that one can get from sexual intercourse. Do you know of anywhere that you can visit to get help in preventing these diseases?

R; I think the best place is the hospital

I; do you know the ways in which they will help you prevent those diseases?

R; I think what they can do is to advise on how you can go about it before engaging in that (sex)

I; In the event of some occurrence and you get pregnant or get infected with some disease at this age, do you know where you can go for help?

R; me privately or with someone?

I; it can be you privately or with someone

R; I will prefer to go to the hospital

I; where exactly in the hospital will you go to?

R; I don’t know where but I will confide in a nurse

I; okay, how useful is the national health insurance to you?

R; I don’t usually fall sick so I don’t use it

I; do you know whether the national health insurance covers some of these services we have been discussing?

R; I am not aware of that

I; Who pays your hospital bills?

R; my parents

I; does the fact that there is money or no money influence your decision to go to the hospital?

R; I can say that if I should fall sick, there is money to take me to the hospital but I don’t use the health insurance. Where I go to hospital, we don’t use the national health insurance

I; you mentioned some changes that are happening in you body to include your menses and mood swings. Have you ever sought for help for any problem regarding these things?

R; I did, and all they keep telling me is it is normal, it is normal, it is normal

I; where did you go for help?

R; I told my mum about it and she told me that as for the mood swings, they are normal and if I should see any change in me, I should just take it as anormal. However, she said if I am to menstruate and it comes for only three days or something, I should let her know because that one is not supposed to be like that

I; do you have any other issues with your menses?

R; I get lots of acne on my face and I get pissed off. I sometimes get pain too

I; have you sought for any help for that?

R; only from my mum

I; apart from your mum?

R; nowhere else

I; what do you do about the painful menses?

R; I just lie prostrate

I; do you take any medication?

R; no

I; why?

R; my mum says it is not good

I; with the acne and the pain, have you ever thought about seeing a health worker or going to the hospital for help?

R; no

I; why?

R; I have been told that it is normal and I have come to accept it as normal

I; have you ever had any infection in your genital area?

R; no

I; have you ever used a contraceptive?

R; no please

I; why not?

R; I am not ready

I; have you ever visited the hospital for any help related to sexual reproductive health or anything of its sort?

R; no

I; in all, does it mean that because you have been told that the things you experience are normal is the reason you don’t seek help for them?

R; no. anytime I feel like there is something changing in me that I need help for, my mum usually has first aid at home so before it gets worse, the first aid reduces the pain so I don’t usually go for check up. The only time I go for check up is when she sees that there are somethings coming on my skin and that is even like once a year

I; what do you think about sexual reproductive health services for adolescents?

R; it is good

I; why do you think it is good?

R; because some of us, we don’t know so if they should come around and educate us it will help us. It will prevent us from getting all these sicknesses and diseases that will isolate us from people

I; do you think there is enough information about these services that will make it easy for adolescents to boldly go and seek help?

R; the problem is that when you go to the hospital, for instance if I should get pregnant at my age and go to the hospital, the way some of the nurses will treat you, you will not even feel like going there again. They will say things like “akola kituaa, won hwemu enko school, woakor nyim. Wobe han yea dwene” (small girl, you wont focus on your education. You have gone to get yourself pregnant and you are coming to worry us). So, no one will feel like going there

I; have you ever experienced this kind of treatment from a nurse?

R; no but I saw it in the hospital being done to someone else. An adolescent girl who was pregnant and had come to the hospital I attended for check up. When it was her turn to see the doctor, she was asleep. So, the nurse came to hit her thigh and said “it is your turn and you are sleeping. Instead of going to school, you have gone to get yourself pregnant”. It was so embarrassing that I was even annoyed

I; so, with what you saw, will you be encouraged to visit the hospital if you find yourself in the same situation?

R; no

I; from these experiences, tell me what you think should be done about these services to encourage young females to become more open to visiting such places to get help for themselves?

R; the service providers should be polite because they do not know what happened that led the young girl to get pregnant. They should be nice and ask what led to the pregnancy. Sometimes, it is not our will to get into such situations. Sometimes they shout at you so much that you start asking yourself why? Like you are in a situation and no one cares about you

R; apart from the staff attitude, what about the facilities?

I; There must be more room for us. Yes, the older women are important but we the adolescents, we need more help. This is because if they don’t teach us, we will go out there and just do anything and not everyone is educated. The illiterates, they will just be there. If you the nurse you don’t help them, they cannot do anything. They have to give us more space and care for us more. The adults have more knowledge and know what to do but we don’t.

I; is there any other information you will want to share with me concerning everything we have discussed so far?

R; I don’t think so

I; thank you very much for participating. I am grateful

R; you are welcome
