# Supplementary material for: Female adolescent sexual reproductive health service utilization concerns: A qualitative enquiry in the Tema metropolis of Ghana
Source: PLoS One. 2024 Feb 23;19(2):e0292103. doi: 10.1371/journal.pone.0292103 (PMC10890758; doi:10.1371/journal.pone.0292103)
Supplement: S1 File — (ZIP) [file pone.0292103.s001.zip › INTERVIEW FOUR TRANSCRIPT.docx]

INTERVIEW FOUR TRANSCRIPT

Interviewer; good afternoon

Respondent; good afternoon

I; how are you doing?

R; I am fine

I; my name is Innes Agbenu, a second-year masters’ student from the university of Ghana school of nursing and midwifery. I am conducting a research on how female adolescents use sexual reproductive health services and this afternoon, as we discussed earlier, we are here to have an interview to discuss issues surrounding sexual reproductive health services for female adolescents. Thank you so much for agreeing to be part of this research and thank you so much for allowing yourself to have this interview with me. Thank you

R; you are welcome

I; first of all, just to start with, can you tell me about yourself?

R; I am fifteen years of age and a third-year secondary school student

I; can you tell me your ethnic group?

R; I am an ewe

I; what religion do you practice?

R; before, I was an atheist but now I am a Christian so I am okay with Christian

I; can you share with me how the change came about?

R; I just moved to school and a whole lot of things happened. I got exposed to the Christian religion in school and I liked the way they do their things so I decided to join and become a christian

I; so, the atheism, was it from your parents, friends, or it was a personal decision?

R; it was from home

I; okay, so your parents are atheists?

R; yes

I; what is your level of education?

R; secondary

I; Tell me about your marital status

R; no, I am not married

I; do you work?

R; no

I; do you have any source of income?

R; mmm, yeah, sometimes

I; what are those sources of income?

R; eerrm, when I go home, sometimes I join competitions, quizzes, a whole lot. Sometimes, I have to help some people I know to make some money

I; so it may not necessarily be official work, but you do some kind of work to earn some income; is that it?

R; yes

I; okay, that’s great. How about your parents? Do you get money from your parents?

R; yes, sometimes, but due to the change of religion, they are not really doing their work as parents

I; can you tell me more about that?

R; well, the moment I changed my mind and told them I wanted to be a Christian, it didn’t really sit well with them. They were angry with me, something I thought will only last for a short while but it turned out to last for two years. We have not been on talking terms for two years now

I; but when you vacate from school do you go home?

R; yeah, sometimes, I go home. At other times too, I go to other relatives but it is not all relatives that are genuine so sometimes, I go to friends. I feel comfortable there

I; are you comfortable going to live with those relatives?

R; yes, very comfortable

I; so, it means that at different times, you live with different people

R; yes

I; Tell me how living with different people at different times feel for you

R; it used to affect my health but it doesn’t bother me anymore

I; you are gradually adjusting?

R; yeah

I; considering your current age, will you classify yourself as a child, an adult or an adolescent?

R; I used to consider myself as an adult because of the things I was going through but I just changed that thought

I; okay, per your age, how will you classify yourself?

R; per my age, adolescent

I; okay. You mentioned earlier that you used to consider yourself as an adult because of the things you were going through. What are some of those things?

R; you see, sometimes I need to do adult stuff in order to earn an income and considering the places, like I changed my location like five times and considering the fact that I have to stay with different people and experience a lot of things, yeah

I; so what are some of those adult things that you do?

R; like, I have to do some works that some adults do

I; like?

R; eeerrm, hawking and some other things

I; you are saying “some other things”. Can you please share what those other things entail?

R; okay, so, sometimes I hawk, sometimes too I work in school canteens, sometimes selling in the night, selling clothes, selling food at other times too

I; does that expose you to any dangers?

R; yes. Because they see you as a child, they try to take advantage of you. One experience that I had, one gentleman thought I was a child so he just wanted to play with me and touch me but I didn’t give him that chance. I had to run away

I; did that cause any problems for you?

R; yeah, he was tracking me and because I couldn’t tell the person I was living with what was going on, so, I had to leave that place and go somewhere else

I; okay, considering everything you have been through and everything you have told me, are there any health needs that you sometimes have that even when you are not sick, you wish you could get help for?

R; no, I don’t like to talk to people because sometimes when you do, they try to trace your parents and talk to them. When they talk to my parents too, they tell lies a lot and I tend to be seen as the bad one. So, I prefer to keep things to myself and if there is anything I need, I know how to get it

I; so, from what you are saying, it means that the needs or problems are there, just that you prefer to keep them to yourself?

R; yes

I; and when that happens, what do you do?

R; sometimes I have to leave school, sometimes, I have to wait till I get a trusted person to get me something that I need for that time

I; alright. As an adolescent, what are some of the changes that are happening in your body?

R; well, I don’t know if these changes are due to adolescence because I have read books and don’t see any of such in them but sometimes, I feel some pains in my chest, at other times too, I check my pressure and it is high. I don’t know if it is because I think too much. Sometimes too, I gain weight. And the menses too, it used to be five days but now it is seven or sometimes eight days, yeah

I; you mean the flow of the menses is up to eight days?

R; yes, first it used to be three to five days but now eight and it comes plenty

I; and what do you do about it?

R; I thought it is just a normal thing so I just need to use the pads and I am off

I; have you spoken to someone about it?

R; well, I see it as normal so I haven’t spoken to anyone about it

I; but you said it started as three to five days and now seven to eight days and from the way you said it, u seem alarmed about. So, have you tried getting any help for it?

R; no ooo.

I; why?

R; well, because it doesn’t come with any pain, I just have to adjust and I haven’t seen anything weird about it so, I haven sought any help

I; I see. What do you know about sexual reproductive health?

R; I think it is about the organs of the body which help in reproduction….. that’s what I know

I; and what else?

R; mmm, that’s all I know

I; and where did you get this information?

R; from school

I; any other place?

R; no

I; okay, as an adolescent at this age, can you share with me some of the things that can happen to you when you engage in sex?

R; obviously, you will get pregnant

I; and what else?

R; If you are not lucky, you might contract some disease
I; okay. So, with this pregnancy and diseases, what are some of the things that you know can be done to prevent them?

R; eermm, maybe, having protected sex or if you know you are not ready, then don’t go for a guy and don’t have sex

I; do you know of any hospitals or clinics where services are provided for adolescents in relation to sexual reproductive health?

R; no but I think when you go to the hospitals, there may be some places like that

I; you think so but you don’t know of anything like that?

R; yes

I; do you know of anywhere that as an adolescent, if you have any issues like with your menses, you have any infections that you can go for help?

R; no

I; you don’t know anywhere that you can confidently walk to for even education or counselling?

R; no

I; do you have any idea what services these places provide?

R; no

I; tell me what you know about contraceptives

R; I think when you use them, it spaces out childbirth or when you don’t want to give birth now, it helps to achieve that. It also helps in family planning. The people who don’t want to get pregnant also use it

I; where did you get this information on contraceptives from?

R; from school

I; apart from school, where else do you get this information from?

R; no please

I; do you know of anywhere that you can go to get help to prevent pregnancy or sexually transmitted infections?

R; in my school, we have a clinic that I know I can go to and in the library too, there are a whole lot of books to read. Some of my teachers too are there for us to talk to

I; do you know of any health facilities that you can go to?

R; no, apart from the clinic in my school

I; we have been talking about preventing pregnancy and sexually transmitted infections, but just in case any of those things happen to you, do you know anywhere that you can go to get help as an adolescent? And when I say infections, I mean both sexually transmitted and non sexually transmitted infections

R; apart from the clinic at school, the hospital will be the other place I will go to

I; do you know of any specific places in the hospital you can go to?

R; no, but I think when you talk to the nurses, they can help you

I; have you heard of adolescent health clinics before?

R; no

I; what about adolescent health corners in hospitals?

R; no

I; have you ever visited the hospital for any issues regarding sexual reproductive health?

R; no

I; you mentioned earlier that you were experiencing heavier menses and longer days of menstrual flow. Did you ever visit the hospital for treatment?

R; no

I; why?

R; I didn’t see it to be so important or necessary

I; what do you think about the national health insurance scheme?

R; hmmmm

I; what do you think about its usefulness towards our health needs?

R; for me, I don’t think it is useful

I; why do you think so?

R; from my experience and the observations I have made with other people too, even when you have the health insurance, you still have to pay huge amounts of money when you need certain medications. It doesn’t show its usefulness

I; okay, if there is a need for you to visit the hospital and you do, who pays the bills?

R; errmm, I do it myself

I; you pay yourself?

R; yes, but fortunately, I don’t visit the hospital often so…

I; so does the availability of money or its unavailability influence your decision to go to the hospital if there is a need for you to go to the hospital?

R; oh yes

I; can you throw more light on that?

R; well, if there is something wrong with me and I need to visit the hospital but I don’t have money, I will hold on and plan before I go to the hospital

I; what if whatever it is you need to visit the hospital for can become a major issue if time is wasted?

R; I will still go

I; have you ever had any issues with your reproductive system that you needed help for?

R; no

I; how about questions on sexual reproductive health that you need answers to?

R; sexual reproductive health doesn’t really bother me so I don’t tend to have questions on it

I; why?

R; I don’t have any problems regarding my reproductive health and I haven’t seen anything I should be scared about so I don’t see the need to know some things

I; if you ever have any questions regarding sexual reproductive health, how will you handle it?

R; I will approach my teachers, especially the ones who will be willing to listen

I; have you ever used a contraceptive?

R; no

I; why?

R; I don’t see the need to use one

I; why haven’t you ever attempted to use any of the adolescent health centers for maybe education or any other service?

R; I don’t know if it is because of my situation but I just don’t like talking to people about things regarding health

I; why?

R; I don’t like it. Sometimes they ask too many questions and sometimes too, they want to know more than that. At times if they feel like you are not giving them the actual information, they may want to talk to your parents. Most of them like doing that

I; what do you think about sexual reproductive health services for adolescents?

R; for those who don’t know anything about it like me who doesn’t like asking people, I think it will be good to have a place where you can go to get information

I; what do you think should be done about sexual reproductive health services for adolescents in order to encourage adolescents to utilise them?

R; actually, it is not everyone you approach with these things that is willing to talk to you and moreover too, the questions are many. There are a lot of things we wish to ask. So, I think it will be good for them to come around and talk to us. Also, when we tell them something, they should do something about it because I have heard others say that when they tell their problems, nothing gets done about it. They should also focus on the individual and their needs

I; do you have adequate knowledge about sexual reproductive health services?

R; personally, I don’t think I have adequate information on it and I don’t think I need adequate information but maybe somebody out there needs it

I; why do you think you don’t need adequate information?

R; I just don’t see the need

I; is there any other information you will want to share with me?

R; no please

I; thank you very much
