# Supplementary material for: Female adolescent sexual reproductive health service utilization concerns: A qualitative enquiry in the Tema metropolis of Ghana
Source: PLoS One. 2024 Feb 23;19(2):e0292103. doi: 10.1371/journal.pone.0292103 (PMC10890758; doi:10.1371/journal.pone.0292103)
Supplement: S1 File — (ZIP) [file pone.0292103.s001.zip › INTERVIEW NINE TRANSCRIPT.docx]

INTERVIEW NINE TRANSCRIPT

Interviewer; good afternoon

Respondent; good afternoon

I; how are you?

R; I am fine. Thank you

I; my name is Innes Agbenu. I am a second-year masters’ student from the university of Ghana. As part of my programme of study, I am conducting a research on how young females utilize sexual reproductive health services within Tema. As we discussed earlier, this afternoon, we are here to have an interview where I will be asking you questions and you will be giving me answers. This interview will be recorded and later written to help us be able to make meaning of whatever you will be telling me concerning the topic under discussion. Thank you once again for agreeing to be part of the research.

R; you are welcome

I; first of all, kindly tell me about yourself

R; my name is spartan, I like sports and I am quick tempered.

I; how old are you?

R; I am sixteen years

I; which ethnic group do you belong to?

R; I am a Ga

I; what is your religion?

R; Christian

I; what level have you gone to school up to?

R; SHS

I; are you married?

R; no

I; do you work?

R; no

I; do you have any source of income?

R; no

I; so how do you get money when you need it?

R; my step dad gives me money for my upkeep

I; whom do you live with?

R; my mum and my step dad

I; with your current age, will you consider yourself as a child, an adolescent or an adult?

R; I am still in my teens so I am an adolescent

I; why do you think you are an adolescent?

R; because I am still within the teen age. The adolescent period is between 10 to 19 years and I am within that age so I am an adolescent

I; at this age, what are some of the things happening to you?

R; I don’t understand

I; considering your current age, what are some of the changes happening in your body?

R; eerrmmm,

I; what are some of the things happening to you now that you didn’t use to experience before?

R; okay, now I menstruate. I didn’t use to menstruate before. There has been an increase in the size of my breast too. Sometimes, if I don’t bath early, I develop some odour

I; with these changes and experiences, are there times you wish to talk to somebody or get some help?

R; when I am menstruating, I feel pains but I don’t take medicine for the pains because I want to endure the pains. I only drink water. Some people take medicine in order to cut the pain. For me, I see the pain as normal

I; so when the pain comes, what do you do?

R; I don’t do anything; I don’t like the painkillers like paracetamol and others that people take

I; do you try going to the hospital for treatment?

R; no but yesterday, I went to the hospital because of pain in my eye and they told me to come back on Saturday so, I will be going

I; did you discuss anything related to your sexual reproductive health with the care providers when you visited the hospital?

R; no, as for those things, my mum will say I should get some herbs to drink so I don’t go to the hospital for them but because my eyes were paining me and I was shouting and couldn’t sleep, they sent me to the hospital yesterday

I; as an adolescent, do you sometimes experience certain things that even when you are not sick, you need some help from the hospital or a healthcare provider?

R; sometime ago, I was dating some guy and he used to call me till my mum got to know abut it. She reacted to it because she didn’t like me seeing that guy and because of that, she doesn’t want me to have a phone. Even as at now, I need a phone because of classes and other information that is sent for my biology classes so I want to talk to someone so that my mum will know that I need the phone and buy it for me or I can buy it from the money I have saved. But my mum still thinks that when you have a phone, you will be doing bad things on the phone she wouldn’t let me use the phone

I; I see. You say you live with your mum and step dad. Are you able to talk to them about your sexual reproductive health issues?

R; no

I; why?

R; hahahaha, my mother? I cannot discuss those things with her. Even when I was sick the other day and I was vomiting she was saying I was pregnant and she was blaming the guy I was dating even though I have never slept with the guy before. Even if I say my stomach or something, she will say I am pregnant.

I; is that why you are not able to talk to her?

R; yes

I; since you are not able to talk to you mother about your sexual reproductive health, do you think of other people that you can be able to talk to?

R; I always talk to my boyfriend

I; do you talk to any other person?

R; no

I; okay, as an adolescent at this age, what do you think are some of the things that can happen to you when you engage in sexual activity

R; right now, at this age, my body is not well developed. My pelvic girdle is not strong enough so if I should get pregnant and try giving birth, it may lead to some effect on my body or may even lead to death.

I; what do you know about sexual reproductive health?

R; physical and mental wellbeing of an individual

I; where did you hear about sexual reproductive health?

R; from school. We were taught in social studies

I; so, what are some of the things that you think can happen to you when you engage in sexual activity at this age?

R; maybe you can get HIV or you can get some fluid from

I; do you know of any adolescent sexual reproductive health clinics around?

R; no

I; do you know of anywhere that young females can go for education and other services related to sexual reproductive health?

R; no

I; have you heard about any services that some clinics provide for young females

R; no

I; do you know ways in which you can prevent pregnancy and diseases that are transmitted from one person to the other through sex?

R; yes

I; can you share with me what you know?

R; I learnt that deep kissing, you will get. Maybe if the person has the disease, you can get. Having sex with many partners too, you can get so you have to avoid having sex with many partners. You have to avoid deep kissing too

I; what else?

R; I also learnt that we have to avoid using sharp objects with other people. If somebody use a sharp object and put it down, we shouldn’t also take it and use. Blood transfusion too, if the blood is infected with HIV, you can also get infected

I; what about pregnancy, which ways do you know in which pregnancy can be prevented?

R; for pregnancy, you have to abstain from sex. Yes

I; have you heard about contraceptives?

R; yes

I; what do you know about contraceptives?

R; I heard that if you have sex and you take it, it washes the sperm out so you won’t get pregnant.

I; do you know some types of contraceptives?

R; yeah, Lydia and postinor 2 or something

I; and which other one?

R; that’s all

I; we have been talking about prevention of pregnancy and diseases transmitted through sex. However, in case something happens and you get pregnant or get any infection which may not necessarily be sexually transmitted but any other infection of the female reproductive system, do you know where you can go to get help?

R; yes, the hospital

I; do you know where exactly in the hospital you can go as an adolescent?

R; no

I; have you ever experienced any infections in your reproductive organs?

R; yes, white. In my house, we have only one toilet and many people are there and everyone uses that place. So, because of that I got white

I; what did you do about it?

R; I told my mum and so they bought some medicine for me

I; do you know the name of the medicine?

R; no

I; did the medicine work?

R; I can’t really tell because sometimes it come and it goes. I think because I still go to use the same toilet so it comes back again

I; so, what are you thinking of doing about it?

R; when I tell my mum, she doesn’t want to take me to the hospital so I tell myself that when I finish school and I start working, I will take myself to the hospital for check ups

I; are you aware that you need to go to the hospital for treatment?

R; yes, I even asked my mum the other day when we went to the hospital that if you come for check up, do you need to go to the consultation place before they teach you where to go? and she said she doesn’t know

I; do you have health insurance?

R; yes, but it is expired. I did it in 2012 and I have never renewed it. I told my mum to renew it for me but she said it will be expensive because from 2012 till now is a long time

I; have you used it before?

R; no, when I did it, I never used it

I; does that mean you don’t know the services it covers or whether it will cover services for sexual reproductive health?

R; it wouldn’t cover it because I have not renewed it

I; if you had renewed it, do you think it would have been useful?

R; yes, because yesterday when I went to the hospital, they asked me if I had it and I said no. so, I paid

I; does the fact that you don’t have money yourself contribute to the reason why you are not going to the hospital for treatment of the white?

R; hmmm, my mother she likes “partey”.

I; what is “partey”?

R; she like going out, buying clothes for funerals and attending parties to have fun so, when I tell her about going to the hospital, she tells me that if I have money myself, I should go

I; if you have money yourself, would you have gone to the hospital?

R; yes

I; who pays when you visit the hospital?

R; my mother

I; does the availability of money or money not being available influence your decision to visit the hospital?

R; yes. She only agreed to take me to the hospital yesterday because I told her I had some money. I will be going to the hospital again on Saturday and I will have to pay 250 cedis so I will have to raise part of the bill myself

I; and where do you get these monies?

R; I get it by saving part of the money my step dad gives me for my upkeep.

I; you were telling me earlier about types of contraceptives. Have you used a contraceptive before?

R; yes. At first, I didn’t know where they have sex through. I thought it was through the clitoris. So, there is this guy whom I attended a party with. He is my boyfriend. During the party, my friend told me that the guy was calling me. When I went to him, he locked the door behind me. I wanted to get out because people were around but he told me that if I go out, people will see that I was inside with him so I stayed inside with him. He tried to have sex with me. Although he tried several times, I was also trying to push him away to stop. So after everything, he told me to go and buy this drug to prevent pregnancy. That was how I took the contraceptive. Later, I asked my friend where they have sex through and she told me what I experienced was the surface but I had already taken the medicine before asking

I; where did you get the medicine from?

R; I bought it at the drug store. After taking the medicine, I have realised that my menstrual cycle has changed

I; when it changed, what did you do about it?

R; I didn’t do anything about it because I think it is because of the medicine I took. I took the medicine because he told me to take it. I later learned that because nothing entered it wouldn’t do anything to me

I; after that time, have you used any contraceptive again?

R; no

I; when your menstrual cycle changed, did you talk to someone about it?

R; no, my sister saw the medicine in my bag and asked me about it and I told her what happened. She asked me why did I attended the party in the first place but I didn’t say anything. I wanted to find out if I am still a virgin because he rubbed his penis on my clitoris and I had some feelings there. So, I wanted to know if I am still a virgin. Anytime I ask, some people tell me yes and some say no. My sister too said it is just the surface

I; do you remember the name of the contraceptive you took?

R; he asked me to go and buy Lydia but when I went, I didn’t get that one. The pharmacist gave me another one

I; do you know of any other place that you could have gone for the contraceptive apart from the drug store?

R; no

I; do you know you could have gone to the hospital for contraceptives?

R; no

I; have you ever been to the hospital for any sexual reproductive health service?

R; no

I; why don’t you visit the hospital for any of those services?

R; I don’t know that when you visit the hospital, you can get someone to talk to about these things

I; in general, what do you think about sexual reproductive health services for adolescents?

R; just like me, I didn’t know when you have sex at the clitoris, it is not actual sex. So, we have to tell others about how these things are including the diseases that are transmitted through sex and how the effects are so that they know

I; so, how do you think these services should be done so that it will be easy for you as adolescents to use them?

R; I think they should send some to schools and hospitals and the community. Also, those who will sit there to talk to us shouldn’t be harsh on us because these days, adolescents, if you talk harshly to them, they will also reply you. The people should be cool and gentle with us

I; is there any other information you will want to share with me concerning everything we have been discussing so far?

R; I want to know if I am still a virgin?

I; I will answer your question after the interview please

R; okay. I don’t have any other information

I; okay. Thank you very much
