# Supplementary material for: Female adolescent sexual reproductive health service utilization concerns: A qualitative enquiry in the Tema metropolis of Ghana
Source: PLoS One. 2024 Feb 23;19(2):e0292103. doi: 10.1371/journal.pone.0292103 (PMC10890758; doi:10.1371/journal.pone.0292103)
Supplement: S1 File — (ZIP) [file pone.0292103.s001.zip › INTERVIEW ONE TRANSCRIPT.docx]

INTERVIEW ONE TRANSCRIPT

Interviewer; good morning, how are you doing?

Response; good morning. I am fine, thank you.

Interviewer; my name Innes Agbenu, a second-year master’s student at the university of Ghana school of nursing. Today, as part of a research I am conduction on the utilization of sexual reproductive health services among adolescent females, we are having this interview to seek your views on the phenomenon under study and also to help as know the needs of female adolescents. Thank you so much for accepting to be part of the study.

Response; you are welcome

Interviewer; to begin with, I will like you to tell me about yourself.

Response; I am a young girl, a secondary school student in form three. I come from Apam in the central region and I live at Ashongman estates.

Interviewer; thank you very much. Please can you tell me your age?

Response; I am 18 years

Interviewer; what ethnic group do you belong to?

Response; I am Fante

Interviewer; please can you tell me about your religion?

Response; I am a Christian

Interviewer; you have already told me you are in form three.

Response; yes

Interviewer; are you married?

Response; no please. I am still a student

Interviewer; do u work or have any source of income?

Response; no please and I don’t have any source of income

Interviewer; okay. Thank you. We will move on to the next phase of questions

Considering your current age, how will you categorise yourself? In terms of a child, an adolescent or an adult?

Response; an adolescent

Interviewer; okay, why will you categorise yourself as an adolescent?

Response; I will categorise myself as an adolescent because maybe I am not at that age that my parents are and I don’t work nor have children to take care of so I am an adolescent

Interviewer; okay. Thank you. Now for you as a female and at this age, tell me some health needs that you have even when you are not sick

Response; okay. I will like to go for check ups because as a student of Tema secondary school, we the students we are many and we use the same washrooms and sometimes infections. And at times you don’t know that you have a particular sickness within you but as you go to the hospital and check your system and your reproductive organs, you get to know whether you have a particular disease

Interviewer; okay, so in essence, you are trying to say that the fact that you are a female and the age group you belong to and where you find yourself, you think that there are some health and reproductive health needs you have even when you are not sick?

Response; yes, yes, yes

Interviewer; okay, lets move deeper into this reproductive health thing and I would want you to tell me about sexual reproductive health. What do you know about sexual reproductive health?

Response; I think it means taking care of your body and not engaging in things that will bring harm to you as a female and as an adolescent

Interviewer; can you tell me where you heard about sexual reproductive health?

Response; okay, we learnt about it in social studies and I got to know that as adolescents, we are not supposed to engage in some activities at this tender age, so we should take care of bodies so that in future we can be able to live peacefully with our families especially our husbands so that we don’t have any issues with our reproductive organs

Interviewer; okay, so you were told about this thing in school?

Response; yes, social studies

Interviewer; so apart from school, have you heard anything about sexual reproductive health outside school?

Response; yes, I learnt that as adolescents, we are not supposed to be in a rush to have sex or engage in some activities because the consequences await us as we grow

Interviewer; so, where did you get this information from? Apart from school?

Response; okay, so friends, not only my age mates but my leaders, pastors, teachers

Interviewer; okay. Share with me some of the things that can happen when you engage in sexual activity at this age

Response; you can easily get pregnant, and also school drop out as a student. It can also lead to prostitution and infections such as STDs and also HIV/AIDS

Interviewer; okay. Let’s move further into the interview. Do you know of any adolescent health clinics in Tema? Or have you heard of any health facilities around Tema that provide services related to sexual reproductive health for adolescents?

Response; no please, I haven’t heard of any such thing. Being in Tema secondary school, if any of such things happen to you, they tell you to go home. For example, if sorry to say you get “white”, they will not tell you to go to a particular hospital or not. They ask you to go home

I don’t think they have such facilities in Tema

Interviewer; okay. Since you say you don’t know about these facilities, do you by chance know what they do?

Response; no please

Interviewer; can you share with me what you know about contraceptives

Response; when I read something about contraceptives, its like when you are pregnant, contraceptives are taken for the baby but I was confused about that because I know that contraceptives are not supposed to be taken as adolescents but I think they just deceive us as if when you have sex and you don’t want to be pregnant, you can take contraceptives but I can say that you can still easily get pregnant even if you use contraceptives

Interviewer; okay. So, where did you learn about contraceptives?

Response; our teachers teach us about our reproductive system and we came across that

Interviewer; what are some of the information that was given to you about contraceptives?

Response; well, I heard contraceptives are there to protect us from diseases and pregnancy

Interviewer; you are a young person, and you have told me about the consequences of engaging in sex at this age. Do you know of ways in which some of these consequences can be prevented?

Response; some girls, it is not that they are willing to engage in sexual activities but because of some family problems like financial problems, that’s why they have to do that so that the men can pay them for them to be able to take care of their school. I think broken homes too….. Please the question again

Interviewer; okay, so, the question I asked was do you know any ways or places where you can get help to prevent pregnancy or infections that can come about as a result of sex because you earlier mentioned to me that when you engage in sex at this stage there are certain things that can come out of it. So, I want to know if you are aware of ways in which to prevent these outcome

Response; I think speaking to our parents about all these things by open up to our parents about why girls engage in these things and maybe I think the doctors too can help maybe because we get the wrong information from friends which make us engage in certain things but when we open up to them, we will be able to get the right information

Interviewer; where are some of the places you can get help?

Response; hospitals, schools, home

Interviewer; so we have been talking about where to go and ways in which to prevent pregnancy and infections associated with sexual activities but in case the pregnancy or infection occurs, or you don’t feel well around your genital area, or you have problems relating to your reproductive health, do you know of places you can go for help?

Response; I don’t know of any place. Okay, maybe the hospital. Apart from the hospital, I don’t know of any other place where you can get help

Interviewer; okay, do you now of any specific places in our hospitals where these services are rendered?

Response; no please. I am just thinking like that. That the hospital should be of help but I have no information about them

Interviewer; how useful is the health insurance scheme to the needs that you have as a female adolescent? You know, you told me earlier that there are certain needs you have as a female adolescent even when you are not sick. How useful is the national health insurance scheme to those needs that you have?

Response; it is very important because, sometimes, maybe if you don’t have money it means you cannot go and get care. Like people who have financial problems, even though they don’t have money, once they have the health insurance, if they visit any hospital, clinic or health center, they will be given some attention

Interviewer; who pays when you visit the hospital? If you are not well and you visit the hospital, who pays the bills?

Response; if you have the card, expenses are not really much

Interviewer; okay, so, as an individual, like you sitting here, if u have to go to the hospital and it comes with a cost, who will pay for it?

Response; my parents

Interviewer; the services that we discussed earlier, considering that those services are not covered by health insurance, will you to go to access those services?

Response; yes, that is if my parents are not having challenges with money. If they have money and I ask, they will let me go

Interviewer; how does money influence your decision to visit the hospital?

Response; quiet

Interviewer; okay, let me rephrase it. Does the availability of money or its unavailability influence your decision to go to the hospital?

Response; yes please

Interviewer; in what way?

Response; like if you don’t have health insurance and you go to the hospital, you may not get the necessary attention and you may have to go home and only go back to the hospital when you get the money. That may even worsen your condition

Interviewer; as a female adolescent, have you ever had any problems with your reproductive system or genital area that you needed help with?

Response; no please. Okay, menstruation and the pain that comes with it

Interviewer; apart from that, what else? You told me that in school, you are many and all of you use the same washroom. So maybe any infections or something

Response; since we are many and use the same washroom, sometimes we get infections like “white”. And the white too is not good because if you don’t go for treatment, it can lead to all sorts of things. Some people say it can let your genital organs smell and you cannot sit in public because the smell can draw people away from you

Interviewer; so, how did you handle these problems when they came? Like the pain with the menses and the white?

Response; with the pain, when it comes, sometimes I sleep. Some adults also advice we use paracetamol so when it becomes severe, I take it. Sometimes too, when I go to the pharmacy, they give me this medicine called “ladinax” and it also helps. Also, with the “white”, some people say we should heat water and put it in a pail and sit on it for a while, and also our panties maybe we don’t wash them well so we should iron them before using. Sometimes too, the doctors and pharmacists prescribe medicines for us

Interviewer; have you ever been to the hospital for the treatment of white or menstrual pain before?

Response; no please, just the pharmacy

Interviewer; in case you develop white now, will you be comfortable going to the hospital?

Response; yes, I will be comfortable because I need to take care of it before it becomes serious. The white is not a good thing or disease to keep around the genital organ

Interviewer; do you know of any specific places in the hospital that you will go for treatment?

Response; no, I don’t know of any specific place

Interviewer; have you ever used a contraceptive?

Response; no, please, I don’t even know how it looks like

Interviewer; have you ever visited any hospital any services related to reproductive health? Like go for information about your body as a young growing woman, or seek information or help on anything related to your body?

Response; no please

Interviewer; does that mean you cannot give me information on the type of facility or how the service providers behaved?

Response; yes please

Interviewer; why haven’t you ever attempted going anywhere for information or service about your body

Response; once I learnt about sexual reproductive health, I thought I had received enough I information on that so I didn’t see the need to see any other person for information

Interviewer; you told me earlier that as a growing young woman, you have needs related to reproductive health even when you are not sick and that sometimes, there might be a sickness in the body that you may not be aware of until you go for check up. Why haven’t you ever tried accessing any help?

Response; I am considering doing that when I finish school because that is when I will leave and not come back here again. So, when I go, and they tell me about any existing problem, they will clear it once and for all. This is because since I am still in school, if they detect any problem and give me treatment, it may come again since I am still in school and we all still use the same washrooms. I don’t want to go back and forth, so I want to complete school then when I go and get treatment, it is once and for all

Interviewer; in this case, do you think that if there is any of such problems already, waiting to finish school before seeking help may prolong the time and lead to worsening of the condition by then?

Response; yes, sometimes I think of that. It is not that I don’t wish to go but it is because of the situation I find myself in now. In this school, they will not allow you to go anywhere except the nurse grants permission. If you see the school nurse and she think the problem is not severe, they will not let you go anywhere

Interviewer; okay. I see. In general, as a society and a country you find yourself in as a young lady growing up, what do you think about how we handle information and services related to young women and their body?

Response; I think they are important to us because once you damage one part, it can lead to a whole lot of problems such as pending a lot of money to solve the problem, frustrations and can even cause you to kill yourself

Interviewer; do you think a lot of awareness creation has been done to help you make decisions regarding your sexual reproductive health?

Response; yes, when you see people such as doctors, nurses and people in these great professions, it encourage you to keep yourself because they tell you that, because they have a future and know what they wanted to become in future, they stayed away from all these things. So, we look up to them

Interviewer; when we talk of sexual reproductive health for women, it involves aspects such as contraceptive services for women, information sharing such as when women visit the hospital, there are places they can confidently go for information, or if a young female gets pregnant, she can confidently go for information and assistance as well as if a young female finds herself infected with any of the STDs, she can boldly go and seek help. These are the things that make up these services. Do you think that these services have been talked about enough such that if you have any of these problems, you can boldly go and seek help?

Response; I think we lack information on such things. I don’t know of anywhere that I can go for information or help that I will feel okay. Also, once you get pregnant, when you go home, you will feel unsafe and they may even sack you from home. I don’t think I can boldly walk to anywhere for help if I get pregnant

Interviewer; what about other things too like infections and other disease?

Response; I will not be comfortable so I will keep it to myself because I don’t have any idea of such places you can visit for such care or even have the courage to talk about it

Interviewer; per what we have discussed so far, share with me how you think these services can be improved in order to encourage young women like you to boldly go and access help whenever you have the need to

Response; I think there should be provision of such services for female adolescents, separate from adults instead of mixing them together. They should create places for only adolescents to come and share the problems. I think this will help because if we share the same place with adults, we will go there and we will even be shy to talk about our problems

Interviewer; okay, so considering that such places do exist, what do you think about information regarding their availability?

Response; I think we lack information about them. That is the reason why even when people get pregnant in school, they try some means of abortion and other things because they do not know where they can go and open up to someone or where they will listen to you. So, what friends say is what you will do even if it is wrong information. I think that with the right information, they will not listen to friends but rather go for help from the right places

Interviewer; okay, thank you very much. Is there any other information you will want to share with me generally based on what we have discussed so far? Anything on your mind that we may not have brought up during the discussion?

Response; I think young females need help especially financial help because that is the main reason why most of them engage in activities that put them at risk of sexual reproductive health issues for example, prostitution. If they are provided with what they need, it will not lead them astray

Interviewer; apart from financial, what other ways do you think adolescent females can get help?

Response; by not listening to friends, and also taking away contraceptives from children. Also, they should be educated properly on sexual reproductive health issues early, so that it will prevent them from doing things that will cause them harm

Interviewer; thank you very much for all the information you have shared with me. We have come to the end of the interview. If there is any other thing, we will get back to you. Thank you very much

Response; you are welcome
