# Supplementary material for: Female adolescent sexual reproductive health service utilization concerns: A qualitative enquiry in the Tema metropolis of Ghana
Source: PLoS One. 2024 Feb 23;19(2):e0292103. doi: 10.1371/journal.pone.0292103 (PMC10890758; doi:10.1371/journal.pone.0292103)
Supplement: S1 File — (ZIP) [file pone.0292103.s001.zip › Interview seven transcript.docx]

Interview seven transcript

Interviewer; good afternoon. My name is Innes Agbenu. I am a second-year masters’ student from the university of Ghana school of nursing and midwifery. As part of the programme, I am conducting a research on how young females within the Tema metropolis utilize sexual reproductive health services. The research seeks to explore their knowledge levels and what influences their decision to utilize sexual reproductive services. This afternoon, as I discussed with you earlier, I am here to have an interview with you where you will be sharing with me information that you have on the topic that we will be discussing. The interview as I mentioned earlier will be recorded and later transcribed in order to help us get the best information. I hope this is okay with

Respondent; yes

I; thank you once again for agreeing to take part in this research. I will be asking you a series of questions and as we go along, we will be discussing a couple of things.

First of all, can you please tell me about yourself?

R; like?

I; in terms of some information about yourself like your age, ethnic group, where you live

R; I don’t know how to start

I; okay, how old are you?

R; I am seventeen years old

I; which ethnic group do you belong to?

R; akyem

I; what is your religion?

R; Christian

I; what is your level of education?

R; junior high

I; are you married?

R; no

I; do you work?

R; no

I; do you have any source of money?

R; yes, like I work but I don’t have any money. They use that money to look after me in the school

I; what work do you do?

R; I sell bags

I; at where?

R; community four

I; is it a shop?

R; yes, it is a big shop

I; are you the owner of the shop?

R; no. It is for my mum

I; oh ok. But you sell for her and make money out of it?

R; yes

I; and you said they use that money to look after you in school?

R; yes

I; whom do you live with?

R; hmmmm, a certain woman

I; you live with a certain woman?

R; yes, all my family members are here but they are not the ones responsible for me as I am talking now. I am just staying with that woman so I can earn some money for food

I; do you live with this woman in the same house?

R; no, she lives at community two and I live with her mum at community four

I; meaning you live with her mum at community four and go to school from there everyday?

R; yes

I; considering your current age, will you describe yourself as a child, an adolescent or as an adult?

R; as an adolescent

I; as an adolescent, what are some of things that are happening to you as an adolescent in terms of changes in your body and thoughts that run through your mind?

R; sometimes, I feel like having sex but I do more to control myself because I know when I have sex and something happens, I don’t have anyone to take care of me so I don’t have sex but I date. I just don’t put more interest in those sexual things like kissing and all those things that will draw me into sex, I don’t involve myself in them

I; okay, what are some of the changes that are happening in your body as an adolescent?

R; okay, sometimes when I am menstruating, I grow fat, I become big but when the menses stop, I go back to normal.

I; what are some of the other things that happen when you are menstruating?

R; I feel pain and I don’t feel like eating. I feel stressed

I; when that happens, what do you do?

R; I just take some painkiller

I; what type of painkiller?

R; gebedol

I; where do you get the painkiller from?

R; I go to the pharmacy and buy

I; have you ever visited the hospital to get help for the menstrual pain?

R; no

I; why?

R; they say it is normal

I; who said it is normal?

R; my mum. She said when I give birth, all these pains will not come again

I; with your current age and the fact that you are a female, do you sometimes have certain needs that although you may not be sick, you think you need help from a healthcare provider or a hospital?

R; yes

I; what are some of those needs? Can you share them with me?

R; at times when I have my menses and I feel stressed, I feel dizzy and I feel like falling. I don’t know what it is but at a certain point, it stops and I fell okay again. Like I said, they said it is normal so I don’t share these things with my mum because she doesn’t like to listen to me since I am not her biological daughter. I don’t like to share my issues with my mum because of that

I; okay, so, this is the person you live with but you are telling me you don’t like sharing things with her. So, who do you talk to when you really need to talk to someone about the things happening to you as an adolescent?

R; I find ways and means to go to the drug store and with my little money, I try to get something. When it is severe, I talk to them that oh mummy, please give me money to seek medical attention so that I can be strong and work for you to enjoy. So, I beg of her and she give me some money and together with my health insurance, I go to the hospital and it makes it a little easy for me to go to the hospital and get medical attention. I at times talk to her and she will help but at times too, she will insult me and send me away or maybe ask me to get some medicine from the pharmacy

I; does that mean that the national health insurance is important to you?

R; yes

I; how does the health insurance help you?

R; some petty petty things, I don’t have to buy them at the hospital. The last time I visited the hospital for an asthmatic attack, they only collected 2 cedis and asked me to go and buy some medicine from the pharmacy which they put on me and I felt better. So, I didn’t pay much

I; have you ever gone to the hospital to get any help related to your sexual reproductive health?

R; no

I; you mentioned that you only talk to the people you live with sometimes when you need help with money to get medical attention, but sometimes, these things are not about money. So, who do you talk to when what you need is not necessarily money but maybe information or counselling concerning your needs as an adolescent?

R; I talk to those who will listen to me

I; have you heard of adolescent reproductive health centres situated within Tema?

R; no

I; so, if you have any need for information concerning your body as a woman especially related to your reproductive organs, who do you talk to?

R; I talk to my friends

I; okay, what do you know about sexual reproductive health?

R; like, when they advise people to avoid sex or take care of themselves

I; what else?

R; nothing

I; you told me earlier on that if you engage in sex at this age, you may have problems. What are some of those things that you think can happen if you engage in sex?

R; I may get pregnant and I don’t want that

I; what else?

R; I may have an infection because I don’t know the kind of person I may end up sleeping with

I; have you heard of any centres in or around Tema that provide sexual reproductive health services for adolescents?

R; no

I; you haven’t heard anyone talk about places in Tema that provide services for adolescents on their sexual reproductive health?

R; no

I; have you by any chance heard about the services they provide?

R; no

I; what do you know about contraceptives?

R; I heard they are pills that those who indulge in sexual activities take so that they don’t get pregnant. It is a drug that help them to prevent pregnancy

I; what are some of the contraceptives you know?

R; I heard gebedol and some others I have forgotten

I; please try to remember

R; they also mentioned Lydia and some others

I; where did you hear about contraceptives?

R; from my friends

I; what brought about that conversation?

R; they are into sexual activities. They always go to “ashawo line” (prostitution) and I am their friend but they always advise me that it is not something that is good so I shouldn’t worry myself and go into it because now that they are in it, it is difficult to stop. They therefore advise me not to get into it. They sometimes tell me that if I am in big big trouble, I can engage in that. Maybe if I need money, I can go to a man and have sex and use that to solve my problems.

I; have you ever tried any of these things they told you?

R; no

I; you told me earlier that if you engage in sex, you can either get pregnant or have some infections. Can you share with me ways that you know can help prevent pregnancy or those infection?

R; yes, they told me that when the man is having sex with you and he feels like “urinating”, that one is the semen so, he has to withdraw. When he withdraws, you won’t get pregnant but it is not safe so I don’t want to try it

I; what else did you hear?

R; they also said that when you sit on the man, that one too, you will not get pregnant. It is like pouring water into a bottle whiles the bottle is turned with the mouth facing downwards. So, when you are pouring the water into it, it will come out

I; apart from these two, do you know of any other methods of preventing pregnancy and infection?

They also mentioned condoms and that there are female condoms that you can wear as a female to prevent that. Even if the man doesn’t wear it, you the female can wear the female condom

I; we have spoken about prevention. But as you know anything can happen. In the event that something happens and pregnancy occurs or you get any infection, do you know of anywhere that you can go for help?

R; no

I; so if you should get pregnant now, what will you do?

R; I will go to my friends

I; why will you go to your friends?

R; because I know when I go to see a nurse, she will not help me abort that child so I will go to my friends. I know that as for them, they have been doing it so they will help me

I; okay. What about the infections?

R; that one I will go to the clinic and see the nurse

I; if you have to go to the hospital, does the fact that there is money or no money influence your decision to go to the hospital?

R; even when I don’t have money, I still go. I go and tell the doctor that the medicine he has written for me, I cannot buy and that I live with somebody so if he can help me. At times, they doctor will tell me to go and that he will come and pay or at other times, he tells the nurses to follow me and help sort it out

I; have you ever had any problems with your reproductive parts apart from the menstrual pain?

R; yes. At times, when I wake up, I feel pains at my vagina area. I feel as if something has happened to me. It stays for sometime before it goes. That is the problem

I; when this happens, what do you do?

R; I don’t do anything about it

I; do you talk to anyone about it?

R; no

I; why?

R; because they mostly don’t listen to me so I don’t like sharing my problems with them

I; have you ever used a contraceptive?

R; no

I; why?

R; because I don’t know what I am using it for. They said it is for sex, so I don’t use it

I; you mentioned that you have never visited the hospital for any services related to sexual reproductive health. Have you any idea how the health workers react when you go to seek help as an adolescent?

R; they will ask you that what did you go to do that you are feeling that pain?

I; how do you know if you have never gone there?

R; I have an idea about it

I; from where?

R; from my friends. A friend is currently facing that problem. So, she has stopped going to the hospital

I; why has she stopped going?

R; she said when she goes, because of her dressing, they ask her where is she coming from and what did she go to do that she is having these problems? They even tell her that she has gone in for abortion that is why she is having those problems

I; in general, what do you think about sexual reproductive health for adolescents?

R; I think we need it. Because many of my friends are suffering a lot but they do not know who to go to

I; what else do you think should be done about these services?

R; I think they should get someone who is nice and can help people get out of their problems. Someone who doesn’t shout at people and who doesn’t frown

I; is there any other information you will want to share with me concerning everything we have discussed?

R; no

I; are you sure?

R; yes

I; okay, we have come to the end of the interview. Thank you so much for participating
