# Supplementary material for: Female adolescent sexual reproductive health service utilization concerns: A qualitative enquiry in the Tema metropolis of Ghana
Source: PLoS One. 2024 Feb 23;19(2):e0292103. doi: 10.1371/journal.pone.0292103 (PMC10890758; doi:10.1371/journal.pone.0292103)
Supplement: S1 File — (ZIP) [file pone.0292103.s001.zip › Interview six transcript.docx]

Interview six transcript

Interviewer; good morning. My name is Innes Agbenu. I am a second-year masters’ student at the university of Ghana school of Nursing and Midwifery. I am conducting a research on how young females use sexual reproductive health services and this morning, we are here to have an interview as discussed. This interview is to allow us discuss issues concerning sexual reproductive health services utilization among female adolescents. I will be asking you a series of questions and your responses will be very important in getting information for this research. The information you will provide will be recorded and later transcribed. I will exclude your name in order to maintain privacy. Thank you so much for agreeing to participate in the study

Respondent; you are welcome

I; first of all, can you please tell me about yourself?

R; I am a student and I lie with my parents here in Tema.

I; how old are you?

R; I am sixteen

I; which ethnic group do you belong to?

R; I am fante

I; what is your religion?

R; I am a Christian

I; are you married?

R; no

I; what is your level of education?

R; senior high

I; are you employed?

R; no please

I; do you have any source of income?

R; no please

I; do you do anything that brings you money?

R; no please

I; so how do you get money when you need it?

R; my parents give me money

I; considering your current age, will you classify yourself as a child, an adolescent or an adult?

R; an adolescent

I; why do you think you are an adolescent?

R; because adolescence ranges from twelve to eighteen years, so I am an adolescent

I; okay, apart from the age, what are some of the things happening to you as an adolescent?

R; there are changes in my body….., yeah

I; what are some of those changes?

R; onset of menstruation, broadening of the hips, development of the breast and everything

I; considering these things happening to you, do you sometimes fell like talking to someone about them?

R; no

I; why?

R; I feel it is something about me and should be with me

I; is that to say there aren’t instances where you feel like you need education on some of the things happening in your body and hence the need to talk to someone?

R; I learn about them in school so, I don’t see any need to see someone for that

I; at this age and with the changes going on, do you sometimes have certain health needs that even though you may not necessarily be sick, you feel you need help from a healthcare provider?

R; yeah, during my menstruation, I get serious pains. I think I have to see a doctor for that. Maybe it is abnormal, maybe it is normal

I; have you seen a doctor for it before?

R; no

I; so, for now, when the pain comes, what do you do?

R; I take medicine for it. Painkillers

I; where do you get the medicine from?

R; pharmacy

I; why haven’t you gone to see a doctor yet for this pain?

R; my mum says it is normal but I don’t see it as normal so I think I need to see a doctor

I; so, when do you intend to see a doctor?

R; when I start living alone

I; when you start living alone?

R; yes

I; so, at the point where you decide to go get this help, do you know where you can go for help?

R; no please

I; have you heard of any adolescent health centres around that you can visit for help?

R; no please, but I know I can visit any hospital. Maybe they can help me

I; tell me what you know about sexual reproductive health

R; it is how to protect yourself sexually, your reproductive system and everything

I; where did you get that information from?

R; school

I; where else?

R; mmm, Tv

I; do you talk to your parents about some of these things?

R; yeah, my mum

I; at this age and with everything happening in your body, what do you think are some of the things that can happen to you when you engage in sexual activity?

R; I can get pregnant and I can get some diseases such as HIV, syphilis and gonorrhea

I; have you heard of any adolescent health clinics around?

R; no please

I; okay, in as much as you may not have heard about them, do you have an idea what services they provide?

R; no

I; the name doesn’t suggest anything to you?

R; they can advise adolescents

I; do you know about contraceptives?

R; yes

I; tell me what you know about contraceptives

R; they are methods that prevent pregnancy during sex. They prevent pregnancy, yeah

I; apart from preventing pregnancies, do you know what else they do?

R; they prevent the spread of diseases

I; do you know about types of contraceptives?

R; yes

I; what are some of the types of contraceptives you know?

R; the condoms and the contraceptive pills

I; where did you get this information from?

R; I was taught in school

I; as a young female, you have shared with me some of the things that can happen if you engage in sexual activity. Do you know of anywhere that you can go to prevent any of those things?

R; mmm, yeah. Hospitals

I; do you know exactly where in the hospitals you can go as an adolescent?

R; no

I; with your age and everything we have discussed so far, should something happen and you find yourself pregnant or develop an infection related to your reproductive system, do you know of anywhere that you can go to get help?

R; no

I; as females, we use washrooms and sometimes get certain infections form that. Have you ever experienced any of that?

R; no

I; how useful is the national health insurance to you?

R; it caters for some of our health fees when we visit the hospital. It takes care of some of the drugs we buy, the services we receive

I; who takes care of your hospital bills?

R; my parents

I; does the fact that there is money or no money influence your decision to visit the hospital?

R; yes

I; so does the fact that you are not earning an income yourself yet influence your decision to go see a doctor for the menstrual pains you have been experiencing?

R; no, money is not one of the reasons

I; apart from the menstrual pain, are there any other things you have ever experienced pertaining to sexual reproductive health?

R; no

I; not even issues with a need for education?

R; sometimes, I want education for that

I; and how did you handle that situation?

R; I went to my mum

I; did she give you the information you needed?

R; yeah …. “Giggles”

I; were you really convinced with what she told you?

R; not really

I; and what did you do then?

R; I searched for information on the net and I still wasn’t so convinced with what I got

I; did you wish you could talk to a healthcare provider?

R; yes

I; do you know there are people like that?

R; yes, I do

I; at where?

R; in hospitals

I; so did you go to talk to any such person?

R; no please

I; why?

R; I can’t go

I; why?

R; I can’t go alone. My mum told me I can’t go there alone

I; and what reason did she give that you can’t go alone?

R; I don’t know. I just can’t go alone

I; there is no reason why you cannot go alone?

R; I don’t have money on me to pay for certain things

I; have you ever used a contraceptive?

R; no

I; why?

R; nothing. I do not think I need it now

I; have you ever visited the hospital for any services related to sexual reproductive health?

R; no please

I; is there any reason why?

R; yes, there is a reason. I am not allowed to go. My mum will not allow me to go

I; in general, what do you think about sexual reproductive health services for adolescents?

R; I think it is a good thing. It will help adolescents know what is going on with their bodies, the changes they experience so that they will not be worried. I think it is a good thing

I; do you think there is enough information on these things to help adolescents patronize them?

R; yeah….

I; do you think there is enough awareness on these sexual reproductive health services for adolescents?

R; at the moment, no

I; why?

R; because we hardly hear things about sexual reproductive health nowadays

I; so tell me what you think can be done to make these services more appealing to you as adolescent

R; they may have to advertise it on Tv, social media and everywhere so that adolescents can also go to health clinics to patronise their services

I; is there any other information you will want to share with me concerning everything we have discussed so far?

R; no please

I; are you sure?

R; yes

I; okay, we have come to the end of the interview. Thank you so much for agreeing to talk to me

R; you are welcome
