# Supplementary material for: Female adolescent sexual reproductive health service utilization concerns: A qualitative enquiry in the Tema metropolis of Ghana
Source: PLoS One. 2024 Feb 23;19(2):e0292103. doi: 10.1371/journal.pone.0292103 (PMC10890758; doi:10.1371/journal.pone.0292103)
Supplement: S1 File — (ZIP) [file pone.0292103.s001.zip › INTERVIEW TEN TRANSCRIPT.docx]

INTERVIEW TEN TRANSCRIPT

Interviewer; good afternoon. My name is Innes Agbenu. I am a student of university of Ghana. I am doing a masters in nursing. I am currently conducting a research on the utilization of sexual reproductive health services among female adolescents in the Tema metropolis. This afternoon, as I informed you earlier, we will have an interview, where I will ask you questions and you will give me answers and we will record it. We will later sit down to write it out for us to be able to know your views on the topic we will be discussing. The information you will give us will be for the purpose of the research only. The information will only be accessed by my supervisors and I only. So, just relax and let’s have a fruitful discussion

Respondent; okay

Interviewer; thank you once again for agreeing to participate in the research

R; you are welcome

I; to start with, please tell me about yourself

R; I am a young girl of 17 years. I am a student. I am very simple and disciplined. I am from a poor background. I am Godfearing and polite to people. I am also fun loving and jovial

I; which ethnic group do you belong to?

R; I am akan

I; from where?

R; oda

I; what is your level of education?

R; SHS

I; are you married?

R; no, I am single

I; do you work?

R; no, I don’t work

I; do you have any source of income?

R; no

I; how do you get money when you need it?

R; from my brother

I; whom do you live with?

R; my brother and my sister

I; where are your parents?

R; they are dead

I; oh ok. Sorry about that. Are the siblings you live with older or younger than you?

R; they are older. My elder brother and sister

I; with your current age, will you categorise yourself as a child, an adolescent or an adult?

R; I am not a child and neither am I an adult. I will say I am an adolescent

I; why do you think you are an adolescent?

R; because I am still in my teen age

I; in this teen age as you said, what are some of the changes happening in your body?

R; my menstrual periods have started coming and development of some areas in my body

I; what are some of those areas?

R; breast and pubic hair. Those things

I; okay, so, with these changes that are happening in your body, do you at times feel like talking to somebody?

R; yes

I; when that happens who do you talk to?

R; I don’t talk to anyone

I; you live with your brother and sister, why don’t you talk to them?

R; I don’t talk to them. I just keep it to myself because I feel like they cannot help me

I; why do you think they can’t help?

R; I used to tell them and they don’t help that’s why I stopped telling them. When I have any issues, I talk to the nurse in my school and if there is any help, she gives it to me

I; do you go to any hospital?

R; no, I don’t go to any hospital

I; why?

R; nothing. I just don’t like going to the hospital

I; why don’t you like going to the hospital?

R; I don’t know. Even when I fall sick, I just stay in the house

I; but why?

R; maybe I am afraid of the drugs and those things that’s why

I; I know you mentioned that even when you are sick, you do not go to the hospital but at this age, are there times that you are not necessarily sick but you have some needs that make you want to talk to a healthcare professional? Do you at times feel that way?

R; yes

I; and what do you do then?

R; I go to the nurse. Any information I need she provides it for me

I; what are some of the issues you go to the nurse for?

R; about my background and my menstrual pains

I; meaning you experience menstrual pains?

R; yes

I; when that happens, what do you do?

R; when it happens, I take paracetamol

I; how many?

R; two, when I take it, it gets a little better. Sometimes too, I sleep until it goes

I; who told you it is okay to take paracetamol for menstrual pains?

R; there was a time I took it and it worked so, I started taking it when I have the pains

I; have you ever visited the hospital to report your menstrual pains?

R; no, there was a time I went to the drug store because I was having menstrual pains and when it comes, I vomit and I can’t walk. They told me that it is something normal and that it will go when I give birth

I; that was what you were told at the drug store?

R; yes

I; have you ever thought about going to the hospital?

R; no

I; what are your thoughts about visiting the hospital for such a problem?

R; I know I can go to the hospital but hmmm, I feel many people experience it so it is normal

I; okay. What do you know about sexual reproductive health?

R; sexual reproductive health?

I; yes

R; things that are going on in our body pertaining to our health

I; where did you get this information from?

R; from my own idea

I; have you ever heard about sexual reproductive health?

R; yes, in social studies

I; and where else?

R; I belong to the adolescent health club and we used to talk about those things

I; okay. Where is this adolescent health club?

R; in school

I; who are the members of this club?

R; we the students and some of the teachers too, so, we used to talk about it

I; what are some of the things they talk about?

R; a lot. Like how to keep yourself, eating well, eating balanced diet and a lot of other things

I; okay, at this age, what are some of the things you know can happen to you when you engage in sex?

R; many diseases like HIV, gonorrhea, syphilis and others

I; apart from the diseases what else can happen?

R; some of the men, some have spirits and they just need you for spiritual things

I; have you heard about teenage pregnancy?

R; yes, I have heard about it. I am afraid ooo. Some of my friends are pregnant and I use that as an advice to myself that if I engage in sexual activity, something may happen to me

I; are you aware of any adolescent health clinics around?

R; no, all I know is the school clinic and the hospitals

I; do you know of any specific places in clinics or hospitals that specifically take care of adolescents?

R; no

I; have you heard of anything like that before?

R; yes, I have heard about that before

I; do you know what they do over there?

R; I don’t know what they do. I have never been to any of them so I don’t know what they do

I; how did you hear about them?

R; when my mother was about to die and we took her to the hospital, my brother drew my attention to a group of people who were my age mates sitting at a place in the hospital going for some service. He told me those are my age mates and that I should join them but I told him I do not have any problems so I wouldn’t go

I; so that was how you got to know that there were clinics for adolescents?

R; yes

I; we were discussing earlier some of the things that can happen when you engage in sexual activity. Like the diseases and pregnancy. Do you know ways in which these things can be prevented?

R; as for the pregnancy, some of them go for family planning and contraceptives

I; what about the diseases?

R; as for the diseases, I know that you will have to go to the clinic to get solutions. I don’t know any medicine that will cure that one

I; as a woman, there are some infections that we get that are not necessarily from sex. Like some genital infections. Have you experienced anything like that before?

R; as for me, mine is “white”

I; when you had it, what did you do about it?

R; I have bought many medications but it is still not going. Just that it is not coming like first. I have realised that anytime I use the school washroom, it comes more

I; where do you get these medicines from?

R; I bought them

I; from where?

R; from the drug store and sometimes, people selling medicines at the roadside

I; have you ever been to the hospital to complain about it?

R; no

I; why?

R; nothing. Just that I feel shy to talk to people about those issues

I; okay, but the white, is it not bothering you?

R; it is bothering me a lot

I; so, what do you plan doing about it?

R; I told my sister and she said she will get medicine for me when she goes to the village so I am waiting for that

I; okay. So, we have been discussing ways to prevent sexually transmitted infections and pregnancy but we also know that sometimes, mistakes happen. In the event that you get pregnant or get any of these infections, do you know anywhere that you can go for help?

R; let me say if I should get pregnant now, I will not abort it. I will rather give birth because I don’t know what the child will do for me in future

I; and what about infections that occur through engaging in sex?

R; with that one too, I will try my best to go to the hospital to get treatment

I; have you heard about contraceptives before?

R; yes

I; tell me about contraceptives

R; what I know is that if you don’t want to get pregnant, you can take it and it will not allow you to get pregnant

I; have you heard about types of contraceptives?

R; yes

I; can you share with me?

R; Lydia, level two and postinor two

I; where did you hear about them?

R; as for Lydia and level two, I heard about them on through advertisement on television but the postinor two, it was just last two weeks that we were discussing something and my friends mentioned it

I; have you used any contraceptive before?

R; I took postinor two when I met a boy who said he will take care of me and he forced me to sleep with me and he bought it for me. I tried to arrest him but he approached my parents to take care of me so since JHS, he has been helping to take care of me.

I; do you live with him?

R; no, I don’t live with him

I; do you still see him?

R; yes, he comes to our place to give me money when my brother doesn’t give me money

I; do you still have some sexual relationship with him?

R; no, apart from that first time, he said that he is waiting for me to finish school so that he will marry me and we will become one

I; the contraceptive that you took, where did you get it from?

R; he bought it for me

I; after that sexual activity, did you go to the hospital for any checks?

R; yes, my sister took me to the clinic and they said there is nothing wrong with me, no disease so I was okay. They just educated me about those things

I; which clinic?

R; lagoon clinic

I; how was the reception when you went there?

R; it was very nice

I; how will you describe how the nurses and doctors behaved towards you?

R; they were very polite and nice

I; how was the setting like in terms of privacy?

R; they received me and spoke to me alone. Not among people

I; how did that impact how you expressed yourself?

R; it made me comfortable to talk to them

I; after that incident, have you taken any contraceptives again?

R; no

I; do you have health insurance?

R; yes

I; have you used it before?

R; no, I did it just last year

I; is it a good thing?

R; they have been saying it is good. My sister had always said I should go and do it and I have not been having time to go and do it. So, last year, I decided to go and do it because I was having a problem with my eye and I have been feeling dizzy. That was why I went to do it

I; whom pays your hospital bills?

R; the guy, he is the one who pays everything when I go to the hospital

I; okay. This guy gives you money and does a lot of things for you as you have said. Doesn’t he sometimes make sexual advances at you?

R; no. he told me never to come to his place. When I complete school, then I can come to his place

I; apart from the menstrual pains and the white you spoke about, have you ever had any problems with your reproductive organs that you needed help for?

R; no

I; so, can you share with me why you haven’t been to the hospital for these problems yet?

R; because I heard it is a normal thing, it will go. My sister used to experience the same thing and after sometime, it stopped so I believe that mine too will go

I; you mentioned that after you took that first contraceptive, you went to the hospital and they gave you some education. Have you ever gone back there again?

R; no

I; why?

R; nothing. I just don’t want to go there again. I feel I don’t have any problem again so there is no need to go there

I; but you complained about the menstrual pains and the white. Don’t you think those are problems?

R; not that I can’t go to the clinic. I can go but my education too is part. I hardly get time for myself after school. That is why I haven’t been able to go

I; I see. What can you say about the fact that you are not working money and your ability to go to the hospital?

R; sometimes, it prevents me from going to the hospital. Sometimes, I feel like because I don’t have money, I should stay in the house and if I have 5 cedis or 10 cedis, I should buy medicine from the drug store or by the roadside

I; if you had money, would you have preferred to go to the hospital?

R; yes, I would have gone to the hospital

I; in general, what do you think about sexual reproductive health services for adolescents?

R; I think it is a good thing and it will be very good if we get educated on that. I think it will help we the adolescents to know our dos and don’ts

I; do you think there is enough awareness on these services to encourage adolescents to utilize them?

R; I don’t think so. If there is information, I don’t think I follow that information. I go to my elders and if there is something, they can tell me, they tell me and I follow it

I; what are some of the things you think can be done to make these programmes better in order to encourage adolescents to utilise them more?

R; they should educate us on how to prevent pregnancy and those things, I think it will help. Most adolescents do no know some of these things. Most of my friends are now pregnant and because I don’t have money, they have been encouraging me to go for boys so that I can get money to care for myself. I just don’t take their advice because I know where I am coming from so I try to live like that

I; do you have any other information to share with me regarding everything we have been discussing so far?

R; I will like to talk to you personally.

I; does it have to do with what we have been discussing so far?

R; some

I; okay, go ahead

R; the contraceptive, does it have any effect on us?

I; okay, I think I will have to answer that after we have ended the interview. Do you have any other information for me?

R; no

I; okay, thank you so much once again for granting me the interview

R; you are welcome
