# Supplementary material for: Female adolescent sexual reproductive health service utilization concerns: A qualitative enquiry in the Tema metropolis of Ghana
Source: PLoS One. 2024 Feb 23;19(2):e0292103. doi: 10.1371/journal.pone.0292103 (PMC10890758; doi:10.1371/journal.pone.0292103)
Supplement: S1 File — (ZIP) [file pone.0292103.s001.zip › INTERVIEW TWELVE TRANSCRIPT.docx]

INTERVIEW TWELVE TRANSCRIPT

Interviewer; good afternoon

Respondent; good afternoon madam.

I; my name is Innes Agbenu, a second-year master’s student from university of Ghana and I am conducting a research on how young females utilise sexual reproductive health services. This afternoon, as I discussed with you earlier, we are here to have an interview. I will be asking you some questions and you will be giving me answers in relation to the topic we will be discussing. That is utilization of sexual reproductive health services among female adolescents. The interview will be recorded and later written out to help us make meaning of what you will be telling me. Thank you once again for agreeing to be part of the research.

First of all, kindly tell me about yourself

R; my name is ice cream. I am seventeen years old and I live with my parents. It’s a long story though. I have been dating a guy for about five years now. For sometime now, all he talks about is sex, sex, sex. So, he told me to go for family planning and I agreed. I did the three years one. So, I still have it

I; which type did you do?

R; it is inside my arm. The type they insert in the arm

I; where did you go to do it?

R; a health center in Ashaiman

I; tell me your experience at the health center when you went to do the family planning

R; when I went, they just asked my name, my age and whether I live with my parents. Then, they told me I am ready for it. They then inserted it into my arm and told me to be coming every month for check up

I; do you go for the check up?

R; yes

I; tell me your experience with the nurses

R; some were plain. They were two, the other one was feeling shy but it was like the first one, she told me everything like I should keep myself, I should stay with only one guy, I shouldn’t go to other men because I think I have done family planning so I can sleep with different men. She told me to be with just one man

I; how will you describe the privacy they provided for you?

R; I was alone with them when I went

I; where you able to express yourself freely when you went?

R; yes

I; what ethnic group do you belong to?

R; I am an Ewe

I; what is your religion?

R; I am a Christian

I; what level have you gone to school up to?

R; senior high school level

I; are you married?

R; no

I; do you work?

R; no

I; do you have any source of income?

R; no

I; so, how do you get money when you need it?

R; I go to my boyfriend

I; what about your parents?

R; they only give me money when it is related to school. If not, when I ask them for money, they tell me we should cook and eat at home. So, when we cook and eat at home, then I go to my boyfriend to give me some money

I; considering your current age, do you consider yourself as a child, an adolescent or an adult?

R; hahaha, as for me, I consider myself as an adolescent

I; why do you consider yourself as an adolescent?

R; now, I can have sex. I can go out. I go out and drink alcohol. I take my decisions myself

I; what are some of the changes happening in your body?

R; just the normal ones like me having my menstruation. Sometimes, I feel pains in my breast, headache. I also feel some emotional things like anger. I feel angry a lot

I; with all these changes, do you talk to somebody about them?

R; no. My parents, they don’t have time for me and if I tell them something like that, they will just say I am becoming a bad girl. That’s what they say

I; so, when it is critical and you really need to talk to someone, who do you talk to?

R; my boyfriend

I; are there times that you have some issues that you wish you could take to the hospital or talk to a healthcare provider even though you are not sick?

R; yes

I; can you share those with me?

R; like this family planning, sometimes, it has been worrying me. It worries me but I don’t know how to tell the doctor. It is not everyday that I can go to where I went to have it inserted. Sometimes, I wish to go to another hospital but I feel shy, like I can’t

I; what are some of these worries that the family planning gives you?

R; sometimes, I feel dizzy a lot and my stomach to has been paining me

I; what was the reason why you inserted the family planning?

R; I got pregnant once and my boyfriend told me I have to abort the baby because he is not ready and me too, I am not ready for that

I; okay, so, what happened to that pregnancy?

R; when I went to where they inserted the family planning for me, I told them everything and the did it for me. After that, they checked and I was not pregnant again before they inserted the family planning for me

I; meaning the pregnancy was aborted for you at the hospital?

R; yes

I; who took you there?

R; my boyfriend

I; are your parents aware of this?

R; no

I; why?

R; I was scared so I couldn’t tell them

I; but you live with them, so, how did you hide all of it?

R; my parents, they don’t have time for me so, they didn’t see anything

I; before then, did you know there was anything you could do to prevent pregnancy?

R; no, my friends have been saying talking about it but I didn’t know what it was until I got pregnant and went to the health center and they told me

I; okay, apart from pregnancy that happened to you out of sexual activity, what else do you know can happen to you?

R; HIV/AIDS

I; apart from HIV/AIDS, what else?

R; that’s what I know, HIV/AIDS

I; what do you know about the family planning you have inserted and HIV/AIDS?

R; mmmm, like how please?

I; what do you know about the family planning you have inserted and prevention of HIV/AIDS?

R; I have realised that even the family planning will let me get HIV easily

I; why?

R; because, this one, it only protects against pregnancy. So, if I have sex with someone who has HIV, I can get it easily

I; so, what are you doing about that?

R; nothing

I; tell me what you know about the ways in which you can prevent HIV/AIDS

R; by using condom

I; so, do you use condom?

R; no

I; why not?

R; hmmmm, my boyfriend will not allow us to use condom

I; have you ever discussed it with him?

R; yes

I; and what did he say?

R; he said no

I; have you ever gone to test for HIV?

R; no

I; what do you think about your possibility of getting HIV or any other disease?

R; I have been thinking about it but I don’t know what I can do about it

I; have you ever tried talking to someone about it?

R; yes, my boyfriend but even that, he just said it is normal, it is normal

I; what do you think about going to a hospital or health center to talk to someone?

R; I have never thought about it

I; have you ever heard about adolescent sexual reproductive health centres?

R; no, I have only heard about adolescent reproductive walk. We go for floats and exercise

I; apart from this walk, you haven’t heard of adolescent reproductive health centres?

R; no.

I; okay, so, when you went to that health center to insert the family planning, what other things did they talk to you about?

R; nothing, they just inserted it for me and told me to stay with one guy

I; what did they say about prevention of HIV and other diseases?

R; nothing

I; did you ask questions when you went?

R; no. At that time, the family planning was what was important to me and I knew that once I do it, I won’t get pregnant so that was all that I was thinking about

I; apart from the family planning you have inserted, what other ways do you know through which you can prevent pregnancy?

R; I know about postinor 2 and the condom

I; okay. The clinic that you went to do the family planning, did you pay for the services?

R; yes

I; who paid?

R; my boyfriend

I; do you have health insurance?

R; yes, I have a private health insurance that I can use at a private hospital

I; okay. As a woman, have you ever experienced any vaginal infection before?

R; yes

I; tell me about it

R; that one, I went to the hospital a couple of times and they gave me medicine but still

I; does that mean it still comes?

R; yes

I; so, what do you do about it?

R; what I do is that I use hot water. I boil hot water and sit on it

I; does that help?

R; not much

I; okay. Tell me about your menstrual experience

R; hmmm, I always feel pain and it does not come nowadays like it used to when I didn’t have the family planning inserted. It does not flow well now

I; what have you done about it?

R; I went to the hospital and the doctor told me it is normal and that sometimes, it will come but this year, I haven’t experienced my menses

I; you mean you haven’t had your menses the whole of this year?

R; yes

I; and what have you done about it?

R; I went to the hospital and they told me it is normal

I; tell me more about the medicines you take for the vaginal infection you said you have been experiencing

R; apart from the hot water, I take the medicines they give me at the hospital

I; okay. On your own, without your boyfriend, are you able to go to the hospital and pay yourself?

R; yes

I; how do you get the money?

R; I save some of the money my parents and my boyfriend give me

I; so, apart from this family planning that you did and going there to report the issues you were having with your menses, have you ever gone to the clinic on your own for any other services?

R; no

I; meaning you only go to clinic with your boyfriend?

R; yes

I; have you ever used any other contraceptive apart from the one you have inserted now?

R; no

I; tell me more about your experience when you went to the hospital to have the pregnancy terminated and the family planning inserted

R; it was cool. It was normal. Everyone was cool with me

I; what do you think about sexual reproductive health services for adolescents?

R; it is good. This is because, some of us go through so many things that we cannot even tell our parents. I think it is good we have somewhere that we can go to talk about our problems

I; so, you think these services are good?

R; yes, it is good. It has helped me so much

I; what do you think about the amount of information available about these services?

R; I think it is good

I; do you think there is enough information available about these services to encourage adolescents to comfortable go and use them?

R; I don’t think there is enough information on these things. It will be good if you can come and help us because our parents, they don’t help us at all. Me like this, I can’t go and tell my mum or dad that I have inserted family planning. I can’t.

I; have you told anyone about the family planning?

R; no. I am shy

I; not even your friends?

R; nobody knows apart from myself and my boyfriend

I; what do you think should be done about these services in order to encourage adolescents who need help like you did to utilise them?

R; I think they should announce it on television. They should have a particular place where they will be so that we can easily go to them. Me like this, if I have some in my area, I will be going more often to get information.

I; okay. Is there any other information you will want to share with me concerning everything we have discussed so far?

R; no

I; are you sure?

R; yes

I; okay. We have come to the end of the interview. Thank you so much for your time. I am grateful. In case of anything, we will get back to you.

R; you are welcome
