# Supplementary material for: Female adolescent sexual reproductive health service utilization concerns: A qualitative enquiry in the Tema metropolis of Ghana
Source: PLoS One. 2024 Feb 23;19(2):e0292103. doi: 10.1371/journal.pone.0292103 (PMC10890758; doi:10.1371/journal.pone.0292103)
Supplement: S1 File — (ZIP) [file pone.0292103.s001.zip › INTERVIEW TWO TRANSCRIPT.docx]

INTERVIEW TWO TRANSCRIPT

Interviewer; good afternoon. I am Innes Agbenu, a student of university of Ghana. I am doing a masters programme in nursing. This afternoon, as part of a research being conducted on the utilization of sexual reproductive health services among female adolescents, I am having this interview with you to help in gathering data. I will be asking you questions on reproductive health and your responses will help in gathering data which will be used in improving those services. Thank you very much for accepting to participate in the study

First of all, I will want you to tell me about yourself

Response; I am a young girl, 18 years, in secondary school, a science student. I don’t do biology. I do geography because I just want to study about the earth.

Interviewer; okay, what ethnic group do you belong to?

Response; I belong to the Ewe side

Interviewer; can you tell me your religion?

Response; I am a Christian

Interviewer; are you married?

Response; no please

Interviewer; Tell me about your work or employment status

Response; I do not work. I am a student only

Interviewer; whom do you live with?

Response; my mum

Interviewer; we will now move into more things about reproductive health. Considering your current age, will you classify yourself as a child, adolescent or an adult?

Response; I am an adolescent

Interviewer; why do you think you are an adolescent?

Response; because the things I used to do as a child, those things have now developed.

Interviewer; any other reason why you think you are an adolescent?

Response; when I was a child, I wasn’t menstruating but now I am menstruating. I am also developing in my body

Interviewer; as a female and at this age, do you think there are some health needs that you have even when you are not sick?

Response; I don’t think so

Interviewer; the question is that, you are a female and an adolescent do you think you have any health needs that even when you are not sick, need some attention? Like issues related to your reproductive health

Response; yes. Like sometimes when we are menstruating, you feel pain and feel weak as if you are sick but you are you are not sick. It is all related to the changes in the reproductive system. Sometimes too, as you develop, you become weak and cannot have the strength as you used to

Interviewer; can you share with me what you know about sexual reproductive health

Response; I don’t really have this thing in it. Like I don’t know how to say it

Interviewer; just say it the way you understand it. You don’t need any technical terms. When you hear the word sexual reproductive health, what comes into your mind?

Response; is it based on the male or female reproductive system?

Interviewer; female

Response; it is based on how the female reproductive system is. Whether fertile or not

Interviewer; okay. Thank you. Can you tell me the source of this information? How you got to hear about sexual reproductive health

Response; I have been hearing about it on television and also, they teach us in our classrooms

Interviewer; okay. So, you have heard about sexual reproductive health from school, on Tv and where else?

Response; sometimes, our parents, when we reach our adolescent age, they tell us that when you do this and that, you will get pregnant so you have to abstain from it

Interviewer; alright. At this age, can you share with me some of the things that can happen to you when you engage in sexual activities?

Response; I can become pregnant

Interviewer; what else?

Response; and if I try to abort, I can die or I can get any disease. It can also lead to early parenthood. Also, if my reproductive organs are not matured for me to give birth, I can get some damages to my reproductive organs

Interviewer; are you aware of any centres located in clinics or hospitals within Tema that provide adolescent sexual reproductive health services?

Response; I don’t actually know anything about that because I am in school here

Interviewer; where you live, do you know of any adolescent sexual reproductive health centers there?

Response; no. they don’t have any

Interviewer; so, generally you don’t know of any centres that provide reproductive health services for adolescents?

Response; no please

Interviewer; well, if you don’t know about those clinics, do you have an idea what services they provide?

Response; they only influence the adolescents to abstain from those things

Interviewer; tell me what you know about contraceptives

Response; its like, maybe you slept with a guy but you don’t want to get pregnant; they are the medicines they sell to us, for you to take so that you don’t get pregnant. Sometimes too, they can fail you. You can take some but still get pregnant

Interviewer; if you want to prevent pregnancy or infections, just like you told me that when you engage in sexual activity at this age, you can get pregnant or some of these infections, where are some of the places that you can go?

Response; you can see a doctor who is qualified in that or you can abstain or you can go to the hospital

Interviewer; do you know of any specific places in the hospital that you can go and get this help?

Response; lets take it like you can go to 37 hospital or Ridge hospital

Interviewer; when you go those hospitals, do you know exactly where you have to go?

Response; no please

Interviewer; if by any chance, you happen to get pregnant now or find yourself with an infection of the reproductive system, where will you go for help?

Response; I will go to the hospital and explain to the doctor

Interviewer; where in the hospital will you go?

Response; first of all, they will give you a place to sit, take your personal details and other things and then send you to the doctor

Interviewer; how useful is national health insurance to you?

Response; it is important because it helps those who don’t have money. When you go the hospital, sometimes the bed you sleep on, you have to pay for it. So, if you have the health insurance, it covers some. If you have to pay full money, if you have the health insurance, instead of the full money, you get to pay half

Interviewer; as a young female, when it comes to sexual reproductive health, do you think the health insurance is important if you ever have to go and access any care at the hospital?

Response; no please, it is not

Interviewer; why do you think so?

Response; I have never experienced it so I don’t know

Interviewer; who pays when you go to the hospital?

Response; my mum

Interviewer; does the availability of money influence whether you go the hospital or not?

Response; if there is no money, maybe she will buy the medicine in the house for me to take to see if I will get better or she will go to the bank for loan so that when she gets money, she will pay later

Interviewer; have you ever experienced any issues with your reproductive organs that you needed help with

Response; yes, like sometimes when I bath with soap and the soap goes there, I feel pain. That’s the only thing I have experienced

Interviewer; you also mentioned earlier that you fell pain during the menses

Response; yes, and I feel pain and feel weak too

Interviewer; you are a student and a lot of you use the same washrooms, do you pick anything from one another?

Response; yes, the last time I even went to the clinic because of that. I was having blood in my bowels. I went home for medicine but when I came back the thing was still happening to me

Interviewer; so how did you handle them?

Response; they gave me a referral to go home and I went to the hospital, and they gave me medicine which I took for one week. I was also instructed not to take any food containing milk nor alcohol. But when I came back to school again, it is still happening

Interviewer; so how are you handling it?

Response; I haven’t been to the hospital again. I haven’t even been to the clinic

Interviewer; have you ever used a contraceptive

Response; no please

Interviewer; have you ever gone to the hospital for any services related to sexual reproductive health?

Response; yes, I was having my menses, and I could change my pads like eight times a day, so, I went to a hospital in Ashaiman. At the hospital, I was told I had lost a lot of blood so I was given injections. I asked the nurse but she didn’t tell me the type of injections

Interviewer; how many times did you go there?

Response; once

Interviewer; did the problem stop after that?

Response; yes

Interviewer; how was the attitude of the people who gave u care?

Response; the doctors were okay but the person who did the test, they were like I was pregnant and it is the pregnancy that is spoilt so they have to do pregnancy test. I didn’t know how to do the test so they gave me a container to go and urinate and I mistakenly urinated for the container to be full. When I brought it back, the man was angry and started insulting me and sacked me to go. Later, he saw me walking with the doctor and called me to come for the results. He thought I had come there by myself.

Interviewer; when he showed you that attitude, how did you feel?

Response; I felt bad but I didn’t react nor talk back

Interviewer; with what happened, will you be able to go back there again in case of another issue and you need help?

Response; I will go but if I am asked to go to where that man is, I will explain to the doctor what he did to me that last time

Interviewer; meaning you will some difficulty if you have to be attended to by that man again

Response; yes please

Interviewer; apart from the injection, did they give you any information on your condition?

Response; they asked me to go their bigger facility for a gynaecologist to check some things

Interviewer; did you go?

Response; when I went home, my mum said she didn’t have money so I didn’t go back

Interviewer; did you ask questions?

Response; I couldn’t ask any questions

Interviewer; why didn’t you ask any questions?

Response; the only question I asked was, they said I was pregnant and I said I should go and tell my mum so I can go and do a womb out wash. But after the test, they realised I was not pregnant but had another problem, that was why the referred me to the gynaecologist

Interviewer; did they give you enough privacy? Did it allow you to feel free and express yourself?

Response; yes, the nurse allowed me to feel free and the doctor too allowed me to tell her what was wrong with me so that she can help me

Interviewer; in general, what do you think about services that are provided for adolescents in relation to their reproductive health?

Response; I don’t know but because our educational system is down, some nurses and doctors do not take their time to explain things to us. Even when you go to the hospital sometimes, they don’t give you the necessary attention, they will be on their phones and they end up giving you a different medicine. You can say you have a headache but they might end up giving you medicine for the stomach. They don’t concentrate

Interviewer; Tell me what you think the health system needs to do to provide sexual reproductive health services specifically for adolescents

Response; Because some adolescents cannot express themselves, because of the way their parents behave, they cannot share their problems with them. So, if they have those services, they can share. Sometimes, when something is happening and I tell my mum, she can say I am a bad girl or something but if those services are there, we will know that even if my mum doesn’t do it for me and I go there, and I tell whoever is there, I will get help

Interviewer; so, you mean those services are necessary?

Response; yes

Interviewer; tell me how these services can be made in order to make them more useful to you as an adolescent

Response; lets take it for instance, my mum hasn’t been to school so if I have some of these problems and I tell her, she will not understand me clearly. If those services are there, those people have been trained so if I talk to them, they will understand. Even though my mum may have experienced it, she will say because of one reason or other, that is why I am feeling that way. Although she may try to understand, because she doesn’t have that knowledge, she may be saying something else

Interviewer; do you think we give young people adequate information about these things?

Response; no please

Interviewer; do you think we can do better? In what ways

Response; by the government helping us in some of those things. We are the next leaders so we have to learn from those who are there

Interviewer; can you share with me any general thoughts on reproductive health?

Response; no please

Interviewer; what are some of the things you think we can do to help you use more of these reproductive health services

Response; like if I tell you something about myself like my problems and you tell other people and I get to know; I may not be encouraged to come back to you for help again and I will also tell my friends not to come to you. Some adolescents too are very shy especially if they have to talk to people they don’t know

Interviewer; thank you for sharing your time and information with me this afternoon. We have come to the end of the interview.

Response; you are welcome
