# Supplementary material for: Female adolescent sexual reproductive health service utilization concerns: A qualitative enquiry in the Tema metropolis of Ghana
Source: PLoS One. 2024 Feb 23;19(2):e0292103. doi: 10.1371/journal.pone.0292103 (PMC10890758; doi:10.1371/journal.pone.0292103)
Supplement: Attachment — Submitted filename: Feedback Plos One Journal 04.02.23.docx [file pone.0292103.s002.docx]

**Feedback on ‘Adolescent sexual reproductive health service utilization concerns: A qualitative enquiry**

**The authors brought out an important issue but the write-up was generally not well written.**

**Title**

Full title: There was no full title and the short title provided did not indicate where the study was conducted and who the study population are. Also, the title is not well written and authors should revise it based on their main objective.

Suggested title: Perceived factors influencing utilization of adolescent sexual and reproductive health service among female adolescents in the Tema Metropolis: A qualitative enquiry.

Abstract

Abstract: Check the grammar under the methods segment. Which metropolis in southern Ghana was the study conducted? Why did they use a semi-structured interview guide to conduct in-depth interviews? There is a difference between content analysis and thematic analysis. The authors should let us know the type of analysis that was done.

The results should contain only the main findings, so the 1^st^ sentence is not required. Factors/barriers to using health services should be properly stated.

Conclusions-The authors should briefly state the key findings and their implications.

Introduction

Please, the authors should edit sentence 57.

Line 74, what initiatives have been undertaken to improve adolescents’ health? What are the effects of these initiatives? Line 83, the grammatical error should be corrected.

84 which metropolis was the study conducted in? Line 84-86, the authors should provide current information about the metropolis. Thus, the introduction should be strengthened with up-to-date statistics since that is readily available. Also, there are several related studies in Ghana but the authors did not include their findings and gaps in previous studies that make the current study relevant.

Methods

The methods are not sufficient enough to replicate this study and for that matter, the authors must expand on it. Which metropolis was the study done in? Where is it located? How many health staff, youth corners etc?

Study setting: This is not well articulated. Sentence 108 should be edited. The Ghana Health Service annual report from the metropolis could be used to expand on why the study is being conducted.

Ethical considerations: A separate heading should be created to discuss ethical issues. Ethical issues should not be blended with procedures for data collection.

Procedures for data collection: How was the in-depth interview guide developed? Was it from literature? Was the tool pre-tested? What language were the interviews conducted in? In all, how many interviews were conducted?

Data Analysis: In the methods, authors talked about content analysis that was performed but in the abstract, they talked about content thematic analysis. They should resolve the discrepancies. They should elaborate on how data was analyzed. Please, edit sentences 131 and 132. What were these codes? Describe the three themes and 12 sub-themes.

Results

This was not well written. Authors should describe the demographic features of the respondents before they present the actual results based on their objectives.

144-149- The themes should be described under the analysis.

Check sentence 152.

Avoid using the names of respondents as you present the results.

There is no full sentence for sentence 188.

Quotes from participants had no full stops.

Authors should avoid describing terms under the results and tell us exactly what the respondents said about those issues.

274-290; the quote is too long. The main issues said by the respondent should be projected.

Discussion

The main findings should be extensively discussed and the implications of the study must stand out.

Conclusions

The implications of the study were not projected.

Study strengths and limitations

These were not projected.

References

The citations are not in line with the requirements of the journal;Vancouver.

Thank you.
