# Supplementary material for: Female adolescent sexual reproductive health service utilization concerns: A qualitative enquiry in the Tema metropolis of Ghana
Source: PLoS One. 2024 Feb 23;19(2):e0292103. doi: 10.1371/journal.pone.0292103 (PMC10890758; doi:10.1371/journal.pone.0292103)
Supplement: Attachment — Submitted filename: Reviewer Comments_PLOS_ONE_2023.docx [file pone.0292103.s003.docx]

**Reviewer Comments**:

**Adolescent sexual reproductive health service utilization concerns: A qualitative**

**Enquiry**

General Impression:

The authors set out to investigate an important and topical policy-relevant subject matter, with a clear aim “The current study explored the predisposing, need, and enabling factors influencing the utilization of sexual reproductive health services among female adolescents in one of the Metropolis in Southern Ghana”, line 35-37. However, the results as presented by the authors do not adequately substantiate the utilisation levels of SRH services among the adolescents. The paper in its current form is also very weak theoretically. Also, there is a disconnect between the results and the discussion, hence the results of the study do not adequately support the conclusion provided.

Abstract:

The abstract is well-written but there are a couple of typos/grammatical errors that appear to derail is flow.

**Major issues**

**Introduction**

**Reviewer comment 1:**

The introduction of the paper is quite OK, and the knowledge gap appears to be well established. However, a major weakness in this section is that given the specifics of the study, the authors needed to situate the work in a clear theoretical/conceptual framework.

**Methods**

**Reviewer comment 2:**

The research design is appropriate for the study. However, the authors mentioned in the abstract in line 38 that a purposive sampling technique was used to select 12 adolescents for interviews. The rationale/justification for the use of purposive sampling and the sample size of 12 participants have not been provided. For example, what informed the choice of 12 participants—why not 20, 8, 40, etc but 12?

**Results:**

**Theme 1: Predisposing factors influencing the utilization of sexual reproductive health**

**services among female adolescents**

**Reviewer comment 3**:

The theme above clearly sets out to explore how various predisposing factors influenced the use of sexual reproductive health services among female adolescents. However, reading through the theme specifically from lines 157-165, it appears the authors have not sufficiently addressed the research question. In particular, in lines 169-181, the authors rather seem to explain the reasons that compel the adolescent girls to engage in sexual activities—for example, financial difficulty (poverty) and not the factors that predisposed them to use SRH services as captured clearly in the study’s aim in line 35 of the paper. The quotes in lines 171-181 do not reflect the preamble/explanation in lines 166-168 under the social factors. The same concerned is observed from lines 184-210.

**Reviewer comment 4:**

Similarly, from lines 211-224, the authors appear interested in establishing the awareness levels of SRH among the adolescents rather than how their knowledge of SRH predisposed them to using the SRH service as the quotes illustrate.

**Reviewer comment 5:**

Again, lines 225-290 appears to explore the adolescents’ source of knowledge about SRH and not how their knowledge about SRH influences their use of SRH services.

**Theme 2: Enabling factors influencing the utilization of sexual reproductive health services among female adolescents**

**Reviewer comment 6:**

While the explanation of the major theme provided by the authors in lines 307-311 is satisfactory, the explanation of the sub-theme “Knowledge of adolescent sexual reproductive health services” lines 312-319 do not support the major theme. Naturally, it would have been interesting to see how the knowledge of the adolescents influenced the use of SRH services or otherwise. The quote in lines 316-319 hardly contributes to the major theme.

**Reviewer comment 7:**

The sub-theme **“**Awareness on sexual reproductive health centers” attempts to determine whether or not adolescents were aware of the existence of reproductive health centres rather than the use of SRH services. The point here is that “awareness” and “utilisation” are distinct variables because the adolescents may be aware of the existence of the reproductive health centres but fail to use them. The focus should have been to establish how the awareness influenced the use of the SRH. The quotes that follow in lines 338-359 do very little to address the major concern regarding the use of SRH services.

**Reviewer comment 8:**

While the sub-themes “Affordability of services” and “Health insurance” appropriately contribute to the major theme above, the quotes in lines 366-377 and lines 379-393 that seem to support these sub-themes do not strongly reflect the major theme. It would have been interesting if the authors provided quotes that directly speak to these sub-themes, they are quite straight forward and easy to comprehend. In addition, the selected quotes need to address the topic directly “use of SRH” services but they (quotes) are generic because in lines 383-384, the authors generally explain the advantages of possessing a valid health insurance as it helps to take care of “petty petty things” and taking care of asthmatic attacks. The fact here is that the authors focus more on the usefulness of health insurance but do not go further to establish how the “usefulness” of health insurance enhances their use of SRH services.

**Theme 3: Need factors influencing the utilization of sexual reproductive health services among female adolescents**

**Reviewer comment 9:**

The authors presented two sub-themes under the major theme above—"Perceived need for health services” line 436 and “View of one’s health” line 458. These sub-themes are actually the same because one’s view of health simply describes the person’s perception about his/her state of health—whether or not people perceive their health problems to be of sufficient importance or magnitude to seek professional health. In fact, the authors’ explanation of the two sub-themes and the narratives/quotes that are selected to support their claims speak to the same issue about the “perceived need” for SRH services for adolescent girls as contained in lines 436-483. In the literature, the two types of need factors that have been identified include—perceived need and evaluated need (see Andersen, 1995; Alatinga et al., 2021).

**Discussion:**

**Reviewer comment 10:**

The major concern in the discussion section relates to the fact that the discussions do not adequately reflect the results. For example, in line 486, the authors categorically stated that “…. revealed poor utilization of sexual reproductive health services among participants” but this claim does not clearly stand out in the results. Again, in line 487, the authors state that “…. Several barriers to utilisation were also identified” yet in the results, the authors only highlighted “financial difficulties” as the major reason for which adolescent do not use SRH services.

**Reviewer comment 11:**

Another major issue in the discussion section is that, the discussions are not anchored on the 3 themes that have been identified in the results. In fact, the discussions have largely been done independent of these themes. For example, in lines 492-497, the authors aver to issues of love, intimacy and belonging, high levels of poverty, etc which are not directly reflected in the results. For example, “In exploring factors that predispose participants to the use of SRH services, it was discovered that in addition to love, intimacy and belonging, most of the participants who were involved in sexual relationships did so for financial gains. This explains why higher poverty, unsafe neighborhoods and poor social support have been identified to be associated with early age of sexual activity initiation and adolescent pregnancies (Decker et al., 2018). All the participants in this study had no source of income but they expressed some financial needs that their parents and guardians are unable to meet,…..”. These explanations in my opinion, are far remote. Plus, granted that adolescents engaged in sexual relationships for financial gains, the authors do not make an impelling case as to how these financial gains enhanced their use of the SRH services or otherwise. For these reasons, there appears to be a disconnect between the themes/results and the discussions.

**Reviewer comment 12:**

The disconnection between the results and discussions is even more pronounced from lines 529-545, where the authors aver to issues of comprehensive sexuality education. In my opinion, this large disconnect between the results and discussions steps from the factor that the paper is not theoretically/conceptually robust. Because while the paper is anchored on the so-called behavioural model and access to medical care—predisposing factors, enabling/inhibiting and need based factors, the relevant literature that discusses this model is not even cited in the entire paper (See for example, Andersen, 1995; Andersen & Newman, 2005; Alatinga et al., 2021). These three references clearly and logically present the three themes of the current paper, and so its theoretical weaknesses result from the fact that the right literature was not consulted to ground it.

**Conclusion**

**Reviewer comment 13:**

The disconnect between the results and the discussions follow through to the conclusion. The conclusion provided in this paper do not adequate emanate from the results—largely, the results do not support the conclusion, and the recommendations provided are very generic and not action-oriented. Because of the disconnect between the results, discussions and conclusion, in my opinion, the paper in its current state adds very little to knowledge in the subject matter, especially in Ghana.

**Minor issues**

**Suggested title:**

**Female adolescent sexual reproductive health service utilization concerns: A qualitative Enquiry in the Tema Metropolis in Ghana**

**Age range of target population**

In the methods section, the authors report that female adolescents aged 14-19 were selected for the study but in the results section, the authors report that adolescents aged 14-18 (line 151) were selected. A reconciliation is needed

**Typos and Grammatical Errors**

The paper will benefit from a general language edit because there are grammatical errors and typos. See lines; 57-58, line 121, lines 140-140, line 185, line 286, line 406, etc

References

The in-text citation in line 66 is not correct. It’s mixed with the journal title.

The reference list needs to be carefully formatted.
