# Supplementary material for: Female adolescent sexual reproductive health service utilization concerns: A qualitative enquiry in the Tema metropolis of Ghana
Source: PLoS One. 2024 Feb 23;19(2):e0292103. doi: 10.1371/journal.pone.0292103 (PMC10890758; doi:10.1371/journal.pone.0292103)
Supplement: Attachment — Submitted filename: Reviewer_comments_2_Ken.docx [file pone.0292103.s005.docx]

Reviewer’s Comments_PLOSONE

The comments have been satisfactorily addressed. However, the manuscript will still benefit from a language edit because there are few minor typos/grammar errors.

1. Under the research design, line 4, page 4, allow should be "allowed"

2. Under the research setting, also line 4 page 4, has should be "had"

3. Under procedure for data collection, line 8 page 5, 12th participants should be 12th participant (singular not plural).

4. Under Demographic characteristics of respondents, line 2 page 6 "three" is missing in the sentence "Nine were senior high school students and THREE were....."

5. Under social factors in page 8 line 3, the sentence "Some adolescents described financial difficulty. They exchange sexual favors for financial" is not complete.

6. The issue of the resistance of the introduction of comprehensive sexuality education in Ghana on page 17 appears very remote to the objectives of the study. Besides, it was not even mentioned in the background/conceptual discussion, hence it appears misplaced in the discussion. I would have deleted.

7. The authors should read the manuscript again and identify the key strengths because just stating that the conceptual framework is its strength, is not robust enough.
