# Supplementary material for: Female adolescent sexual reproductive health service utilization concerns: A qualitative enquiry in the Tema metropolis of Ghana
Source: PLoS One. 2024 Feb 23;19(2):e0292103. doi: 10.1371/journal.pone.0292103 (PMC10890758; doi:10.1371/journal.pone.0292103)
Supplement: Attachment — Submitted filename: Feedback for Authors.docx [file pone.0292103.s006.docx]

**Female adolescent sexual and reproductive health service utilization concerns: a qualitative enquiry in the Tema Metropolis in Ghana.**

Generally, authors have improved upon the write-up. However, there are still some issues that require redress. The comments below should be addressed to strengthened the quality of the article. Sexual Reproductive Health (SRH) should always be defined as sexual and reproductive health.

**ABSTRACT**

Background Information

Authors could add “…and yet evidence on utilization of SRH services among female adolescents is sub-optimal”.

Methods

Authors should check the difference between semi-structured interviews and unstructured interviews. Also, there is a difference between thematic analysis and thematic content analysis.

Results: Since this is a qualitative study, authors should indicate “that **perceived** utilization of SRH services was low”.

**Conclusion**

The last sentence of the conclusion has no full stop. **Perceived** **factors** of utilization of SRH should be captured under the conclusion.

**ARTICLE**

**Introduction**

Sexual reproductive health services (SRHS) should be written as Sexual and Reproductive Health Service. Adolescent Sexual Reproductive Health should also be captured as Adolescent Sexual and Reproductive Health.

Worldwide adolescents face numerous challenges…… Authors should also add the challenges faced by adolescents in the African context including Sub Saharan Africa before giving us the picture in Ghana.

The study focuses on utilization of sexual and reproductive health services among female adolescents and perceived factors of utilization. But under the introduction, I have not seen any previous related evidence around this area. There is so much published evidence around it but authors did not include any of it. For instance, what related works have been done elsewhere including Ghana about the topic? What are the gaps in these related works that the study seeks to address?

The Anderson and Newman behavioral health services utilization model was used…. Sentences about the model were not referenced at all. All factual statements made by authors should have citations. Also, the sentence about the model should be put at an appropriate location. Where it is currently does not make the reading of the article interesting.

**Study** **setting**

There are factual statements without citations. Authors should fix them.

**Data Collection Procedures**

The data collection tool should be provided.

Interview guides were developed based on the constructs of the conceptual model used, reviewed literature… Provide citation. But the conceptual model being referred to is rather a theoretical model.

**Findings**

Authors should put the explanation below under data collection procedures. The results aspect of the article is only meant for presentation of results;

‘Predisposing factors refer to factors that influence either positively or negatively utilization of services. Enabling factors are factors that make utilization of services easier. Need factors are situations that propel an individual to service utilization’.

Demographic characteristics

‘Nine were senior high school students and were junior high school graduates’. Authors should show the table for the demographic features.

There should be a preamble or an appropriate topic sentence or heading before introducing the Table 1.

Authors should critically look at the themes and sub themes again. Those that are not really perceived factors of utilization of SRH should be removed. Also, I have not seen a theme on perceived utilization of SRH. Under the results, authors said it was low but I have not actually seen detail findings on it.

**Discussion**

The discussion segment should be focused on main findings on utilization of SRH services and the perceived factors. But other issues not related to the study objectives were discussed. Please, correct **‘unwanated consequence’.**

**Conclusions**

… The study revealed low utilization of SRH services...Add the full stop to the last sentence.

**Strengths and limitations of the study**

This should be strengthened because what is currently captured is inadequate.

**References**

Some citations are Vancouver whilst others are not. The journal uses the Vancouver referencing style and so authors should revise citations based on this recommended style. The reference list should be reorganized.
