# Supplementary material for: Female adolescent sexual reproductive health service utilization concerns: A qualitative enquiry in the Tema metropolis of Ghana
Source: PLoS One. 2024 Feb 23;19(2):e0292103. doi: 10.1371/journal.pone.0292103 (PMC10890758; doi:10.1371/journal.pone.0292103)
Supplement: Attachment — Submitted filename: Responses to reviewers comments -2.docx [file pone.0292103.s007.docx]

Title: **Female adolescent sexual reproductive health service utilization concerns: a qualitative enquiry in the Tema Metropolis of Ghana.**

RESPONSES TO REVIEWERS COMMENTS

3^rd^ May, 2023

|  | **REVIEWER’S COMMENTS** | **AUTHORS RESPONSES** |
| --- | --- | --- |
| 1. | Please amend the title either on the online submission form or in your so that they are identical. | This has been corrected |
| 2. | Your ethics statement should only appear in the Methods section of your manuscript. If your ethics statement is written in any section besides the Methods, please move it to the Methods section and delete it from any other section. Please ensure that your ethics statement is included in your manuscript, as the ethics statement entered into the online submission form will not be published alongside your manuscript. | The ethics statement is captured only under “ethical consideration” in the methods section. |
