# Supplementary material for: Female adolescent sexual reproductive health service utilization concerns: A qualitative enquiry in the Tema metropolis of Ghana
Source: PLoS One. 2024 Feb 23;19(2):e0292103. doi: 10.1371/journal.pone.0292103 (PMC10890758; doi:10.1371/journal.pone.0292103)
Supplement: Attachment — Submitted filename: RESPONSE TO REVIEWER COMMENTS(1).docx [file pone.0292103.s004.docx]

**RESPONSE TO REVIEWER COMMENTS**

**MANUSCRIPT TITLE**: Female adolescent sexual reproductive health service utilization concerns: A qualitative enquiry in the Tema Metropolis in Ghana.

| **REVIEWER 1 COMMENTS** | **RESPONSE FROM AUTHORS** |
| --- | --- |
| 1. Full title: There was no full title and the short title provided did not indicate where the study was conducted and who the study population are | Thank you for your comment  The title of the study has been revised to:  “Female adolescent sexual reproductive health service utilization concerns: A qualitative enquiry in the Tema Metropolis in Ghana”. Kindly find the revised title on Page 1 |
| 2. Abstract: Check the grammar under the methods segment.   Which metropolis in southern Ghana was the study conducted?  Why did they use a semi-structured interview guide to conduct in-depth interviews?  There is a difference between content analysis and thematic analysis. The authors should let us know the type of analysis that was done.    The results should contain only the main findings, so the 1st sentence is not required. Factors/barriers to using health services should be properly stated. Conclusions-The authors should briefly state the key findings and their implications. | All comments on the abstract have been well noted and addressed as;  Grammar under the methods segment has been checked and corrections made. Page 2  The metropolis in which the study was conducted (Tema metropolis) has been added to the write up.  Semi-structured interview guide was used to provide guide questions for the interviews but also give room for participants to express themselves in details given the sensitive nature of the topic and allow the researchers to probe for further details where necessary  This was a genuine typo error and has been rectified. Data analysis was by content analysis with details provided in the write up.  The results section has been revised to contain only the main findings of the study. Factors/barriers to utilization have been stated  The conclusion has been revised to state the key findings and the implications of the study.  Kindly find the above revisions on page 2 |
| 3. Introduction Please, the authors should edit sentence 57. Line 74, what initiatives have been undertaken to improve adolescents’ health? What are the effects of these initiatives? Line 83, the grammatical error should be corrected. 84 which metropolis was the study conducted in? Line 84-86, the authors should provide current information about the metropolis. Thus, the introduction should be strengthened with up-to-date statistics since that is readily available. Also, there are several related studies in Ghana but the authors did not include their findings and gaps in previous studies that make the current study relevant. | These comments have been well received and  Sentence 57 has been edited    Also, the initiatives undertaken to improve adolescent health have been added to line 74  The grammatical error in line 83 has been corrected  The name of the metropolis has been added to line 84  Current information has been provided about the metropolis. Statistics on adolescent health in the metropolis have been added to the write up.  These revisions can be found on Page 3 to 4 |
| 4. The methods are not sufficient enough to replicate this study and for that matter, the authors must expand on it. Which metropolis was the study done in? Where is it located? How many health staff, youth corners etc?   Study setting: This is not well articulated. Sentence 108 should be edited. The Ghana Health Service annual report from the metropolis could be used to expand on why the study is being conducted.  Ethical considerations: A separate heading should be created to discuss ethical issues. Ethical issues should not be blended with procedures for data collection.  Procedures for data collection: How was the in-depth interview guide developed? Was it from literature? Was the tool pre-tested? What language were the interviews conducted in? In all, how many interviews were conducted?  Data Analysis: In the methods, authors talked about content analysis that was performed but in the abstract, they talked about content thematic analysis. They should resolve the discrepancies. They should elaborate on how data was analyzed.  Please, edit sentences 131 and 132. What were these codes? Describe the three themes and 12 sub-themes. | Comments well noted  Information on the metropolis where the study was conducted, the location, details about the number of health staff and youth corners have been provided in the paper  Details on the study setting has been updated. Information on the Ghana Health service annual report on the Tema metropolis has been used to make a case for the relevance of the study to the metropolis  A separate heading has been created to discuss the ethical issues  Information on how the interview guide was developed and pre-test and have been updated  Interviews were conducted in English and 12 interviews were conducted in all, all these have been provided in the paper  This has been resolved and details on how data was analyzed have been provided.  Page 4 to 6 contains the above revisions  This has been revised. Descriptions have been provided for the themes and sub themes  Page 4 to 6 |
| 5. Results This was not well written. Authors should describe the demographic features of the respondents before they present the actual results based on their objectives. 144-149- The themes should be described under the analysis. Check sentence 152. Avoid using the names of respondents as you present the results. There is no full sentence for sentence 188. Quotes from participants had no full stops. Authors should avoid describing terms under the results and tell us exactly what the respondents said about those issues. 274-290; the quote is too long. The main issues said by the respondent should be projected | The authors have taken note of these comments and the following actions have been taken;  The demographic features of the respondents have already been described in the paper  Description for the themes have been done  The names used are pseudo names and not the actual names of participants  Full stops have been added to participant quotes.  Quotes have been reviewed and shortened  Page 6 to 15 |
| 6. Discussion The main findings should be extensively discussed and the implications of the study must stand out. | The discussion has been revised to project key findings and implications on page 16 to 18 |
| 7. Conclusions The implications of the study were not projected. | This has been resolved with implications added to the conclusion on page 18 |
| 8. Study strengths and limitations These were not projected. | The strengths and limitations of the study have been added. Page 18 |
| 9. References The citations are not in line with the requirements of the journal; Vancouver. | Citations have been changed from APA to Vancouver. Page 19 to 20 |

| **REVIEWER 2 COMMENTS** | **RESPONSE FROM AUTHORS** |
| --- | --- |
| 1. Abstract: The abstract is well-written but there are a couple of typos/grammatical errors that appear to derail is flow. | Thank you for your comments.  Comments on the abstract have been reviewed with typos/grammatical errors corrected on page 2 |
| 2. The introduction of the paper is quite OK, and the knowledge gap appears to be well established. However, a major weakness in this section is that given the specifics of the study, the authors needed to situate the work in a clear theoretical/conceptual framework. | Comment well noted and addressed.  The Anderson and Newman Behavioural model for health service utilization was used to guide the study and has been updated as such.  Kindly access this revision on page 3 to 4 |
| 3. The research design is appropriate for the study. However, the authors mentioned in the abstract in line 38 that a purposive sampling technique was used to select 12 adolescents for interviews. The rationale/justification for the use of purposive sampling and the sample size of 12 participants have not been provided. For example, what informed the choice of 12 participants—why not 20, 8, 40, etc but 12? | The comments have been well noted and the rationale and justification for the sampling technique and sample size have been updated.  These can be found on Page 4 to 5 |
| 4. Theme 1: Predisposing factors influencing the utilization of sexual reproductive health services among female adolescents  Reviewer comment 3: The theme above clearly sets out to explore how various predisposing factors influenced the use of sexual reproductive health services among female adolescents. However, reading through the theme specifically from lines 157-165, it appears the authors have not sufficiently addressed the research question. In particular, in lines 169-181, the authors rather seem to explain the reasons that compel the adolescent girls to engage in sexual activities—for example, financial difficulty (poverty) and not the factors that predisposed them to use SRH services as captured clearly in the study’s aim in line 35 of the paper. The quotes in lines 171-181 do not reflect the preamble/explanation in lines 166-168 under the social factors. The same concerned is observed from lines 184-210. | The authors have taken note of this comment and have revised the results to project the various predisposing factors and how they influenced utilization of SRH services.  This is on Page 8 |
| 5. Reviewer comment 4: Similarly, from lines 211-224, the authors appear interested in establishing the awareness levels of SRH among the adolescents rather than how their knowledge of SRH predisposed them to using the SRH service as the quotes illustrate. | This has been addressed to reflect what the quotes illustrated. Page 12 |
| 6. Reviewer comments 5 to 7 were pointing to how theme 2 and its sub-themes were presented | The themes and sub-themes mentioned in these comments have been revised to reflect how these factors influenced utilization of SRH services among female adolescents.  Page 11 to 15 |
| 7. Reviewer comment 8. Reviewer comment 8: While the sub-themes “Affordability of services” and “Health insurance” appropriately contribute to the major theme above, the quotes in lines 366-377 and lines 379-393 that seem to support these sub-themes do not strongly reflect the major theme. It would have been interesting if the authors provided quotes that directly speak to these sub-themes, they are quite straight forward and easy to comprehend. In addition, the selected quotes need to address the topic directly “use of SRH” services but they (quotes) are generic because in lines 383-384, the authors generally explain the advantages of possessing a valid health insurance as it helps to take care of “petty petty things” and taking care of asthmatic attacks. The fact here is that the authors focus more on the usefulness of health insurance but do not go further to establish how the “usefulness” of health insurance enhances their use of SRH services. | The sub-theme health insurance had a participant quote which reflected how useful the health insurance is to the utilization of some SRH services for adolescents. Page 13 |
| 8. Reviewer comment 9.  The authors presented two sub-themes under the major theme above—"Perceived need for health services” line 436 and “View of one’s health” line 458. These sub-themes are actually the same because one’s view of health simply describes the person’s perception about his/her state of health—whether or not people perceive their health problems to be of sufficient importance or magnitude to seek professional health. In fact, the authors’ explanation of the two sub-themes and the narratives/quotes that are selected to support their claims speak to the same issue about the “perceived need” for SRH services for adolescent girls as contained in lines 436-483. In the literature, the two types of need factors that have been identified include—perceived need and evaluated need (see Andersen, 1995; Alatinga et al., 2021). | This comment has been well noted. However;  The two sub-themes were based on the conceptual framework used. Viewpoint of one’s health was explained in terms of the severity one attaches to a health condition which will necessitate health service utilization whiles the need for health service was explained in terms of whether or not an individual sees a given health service worthy of utilization. This is because, an individual may hold a viewpoint that their health problem needs attention but may not necessarily think that this attention should come from a health service. Hence the reason some people may patronize alternative treatment options. Page 14 to 15 |
| 9. Reviewer comment 10-12. Reviewer comment 10: The major concern in the discussion section relates to the fact that the discussions do not adequately reflect the results. For example, in line 486, the authors categorically stated that “…. revealed poor utilization of sexual reproductive health services among participants” but this claim does not clearly stand out in the results. Again, in line 487, the authors state that “…. Several barriers to utilisation were also identified” yet in the results, the authors only highlighted “financial difficulties” as the major reason for which adolescent do not use SRH services.  Reviewer comment 11: Another major issue in the discussion section is that, the discussions are not anchored on the 3 themes that have been identified in the results. In fact, the discussions have largely been done independent of these themes. For example, in lines 492-497, the authors aver to issues of love, intimacy and belonging, high levels of poverty, etc which are not directly reflected in the results. For example, “In exploring factors that predispose participants to the use of SRH services, it was discovered that in addition to love, intimacy and belonging, most of the participants who were involved in sexual relationships did so for financial gains. This explains why higher poverty, unsafe neighborhoods and poor social support have been identified to be associated with early age of sexual activity initiation and adolescent pregnancies (Decker et al., 2018). All the participants in this study had no source of income but they expressed some financial needs that their parents and guardians are unable to meet,…..”. These explanations in my opinion, are far remote. Plus, granted that adolescents engaged in sexual relationships for financial gains, the authors do not make an impelling case as to how these financial gains enhanced their use of the SRH services or otherwise. For these reasons, there appears to be a disconnect between the themes/results and the discussions.  Reviewer comment 12: The disconnection between the results and discussions is even more pronounced from lines 529-545, where the authors aver to issues of comprehensive sexuality education. In my opinion, this large disconnect between the results and discussions steps from the factor that the paper is not theoretically/conceptually robust. Because while the paper is anchored on the so-called behavioural model and access to medical care—predisposing factors, enabling/inhibiting and need based factors, the relevant literature that discusses this model is not even cited in the entire paper (See for example, Andersen, 1995; Andersen & Newman, 2005; Alatinga et al., 2021). These three references clearly and logically present the three themes of the current paper, and so its theoretical weaknesses result from the fact that the right literature was not consulted to ground it. | These comments have been well noted.  In response to these comments, the discussion has been updated to address the issues raised. The literature concerns mentioned by the reviewer has also been addressed. These are on Page 16 to 18 |
| 10. Reviewer comment 13.  The disconnect between the results and the discussions follow through to the conclusion. The conclusion provided in this paper do not adequate emanate from the results—largely, the results do not support the conclusion, and the recommendations provided are very generic and not action-oriented. Because of the disconnect between the results, discussions and conclusion, in my opinion, the paper in its current state adds very little to knowledge in the subject matter, especially in Ghana. | The conclusion section of the paper has also been revised to address the concerns raised by the reviewer. Page 18 |
| 11. Minor issues raised. Suggested title: Female adolescent sexual reproductive health service utilization concerns: A qualitative Enquiry in the Tema Metropolis in Ghana  Age range of target population In the methods section, the authors report that female adolescents aged 14-19 were selected for the study but in the results section, the authors report that adolescents aged 14-18 (line 151) were selected. A reconciliation is needed  Typos and Grammatical Errors The paper will benefit from a general language edit because there are grammatical errors and typos. See lines; 57-58, line 121, lines 140-140, line 185, line 286, line 406, etc  References The in-text citation in line 66 is not correct. It’s mixed with the journal title. The reference list needs to be carefully formatted. | Thank you for all the comments. The minor issues raised have been addressed as such;  The title has been revised. Page 1  The age range of the participants has been reconciled. Page 1  The typos and errors have been edited. Page 1  The references have been formatted to Vancouver style.  All these revisions can be found on Page 19 to 20 |
